# Shifts and expansion in a spatially structured fish stock spanning international borders in the Northwest Atlantic

**DOI:** 10.1002/eap.70311

**Published:** 2026-09-16

**Authors:** Nancy L. Shackell, Kiyomi J. Ferguson, Andrew J. Allyn, Cornelia E. den Heyer, Jamie C. Tam, David M. Keith, Katherine E. Mills, James T. Thorson

**Affiliations:** ^1^ Fisheries and Oceans Canada, Bedford Institute of Oceanography Dartmouth Nova Scotia Canada; ^2^ Gulf of Maine Research Institute Portland Maine USA; ^3^ Habitat and Ecological Processes Research Alaska Fisheries Science Center Seattle Washington USA

**Keywords:** Atlantic halibut, distribution shifts, fisheries, integrated modeling, species distribution modeling, transboundary

## Abstract

Marine commercial fish species can shift their geographic distributions as environmental conditions change. Distribution shifts of commercial species across borders pose a challenge because individual jurisdictions often conduct stock assessments using data from within their own borders only. This can lead to fisheries managers reacting to local trends in abundance that do not represent stock‐wide status. Atlantic halibut (*Hippoglossus hippoglossus*) spans across Eastern Canada and extends into the United States, across a French territory and into the High Seas. Here, we integrate survey data from the United States and Canada to test for distribution shifts across Atlantic halibut's range. In Canada, the halibut fishery is certified as sustainable, whereas in the United States, the same population is designated as “overfished”. We used a spatiotemporal modeling approach to integrate US and Canadian fisheries‐independent surveys so as to examine a suite of distribution shift indicators at both regional and smaller (focal area) scales, before and during a period of enhanced warming (1990–2005 and 2006–2023, respectively). Abundance increased in both the United States and Canada over the full time series. The proportion of halibut abundance in Canada ranged from 89 to 96%, averaging 94% over the time series. As abundance increased during the 2006–2023 period, the center of gravity (COG) shifted at a rate of 4 km/year north (95% CI: 3–4.3 km), and 12 km/year east (95% CI: 10–15 km) in Canada. During the same period in the United States, the COG shifted southwest at 3 km/year south (95% CI: 1.5–4 km) and 1 km/year west (95% CI: 0.5–2 km). As the population increased between 2006 and 2023, the leading edge of the range expanded northward and predominantly eastward ~270 km, while the trailing edge shifted northward and predominantly eastward 70 km, consistent with the shelf topography and overall northern expansion. Our results are consistent with the spatial expansion of suitable thermal habitat and an increase in the length of the growing season for Atlantic halibut throughout the region. Here, we recognize the need to operationalize distribution shift indicators for fisheries management and other fisheries‐focused decision‐support frameworks.

## INTRODUCTION

Global warming has already had a profound impact on the geographic distribution of a wide range of plants and animals, most notably in rapidly warming areas, as species move to more suitable habitat (Chen, 2011; Parmesan, 2006; Pecl et al., 2017). The knowledge, detection, and monitoring of changes in species distributions driven by ecological and environmental drivers are an integral component of natural resource management, particularly in an era of climate change. This is especially true in the ocean, where marine ectotherm distributional shifts are more closely aligned with their thermal tolerance limits than their terrestrial counterparts (Sunday et al., 2012). Marine species distribution shifts are occurring more rapidly (Lenoir et al., 2020; Poloczanska et al., 2013), and an impact on fisheries has been detected through the average preferred temperature of commercial species, which has increased globally (Cheung et al., 2013). Incorporating the impacts of climate change into fisheries management has become a global imperative and, in North America, has been highlighted in changes to Canada's Fisheries Act (Bill C‐68, 2019), and the implementation of the United States' Magnuson‐Stevens Act (Procedure for Addressing Climate Change in NMFS Essential Fish Habitat Consultations). Efforts by both Canada and the United States include commitments to an ecosystem approach to fisheries management (Ecosystem approach to fisheries management (EAFM); Northeast Ecosystem and Socioeconomic Profile Workshop Report) which supports sustainable fisheries and adaptive management.

**FIGURE 1:**
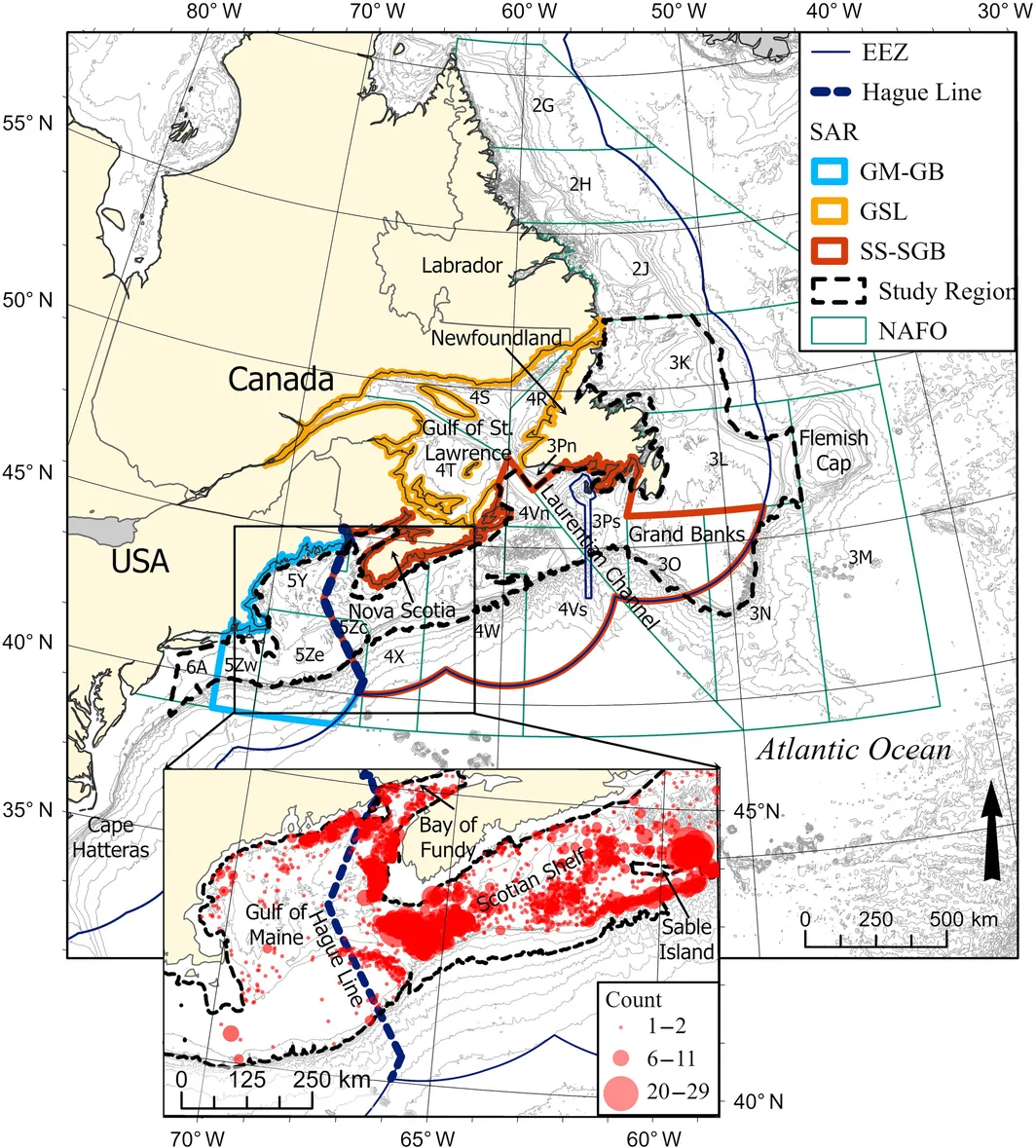
Northwest Atlantic Ocean. The study region (dashed black line) for this analysis represents the range of Atlantic halibut (*Hippoglossus hippoglossus*) in the United States (NOAA NEFSC) and Canada (DFO Maritimes and Newfoundland) covered by research vessel (RV) surveys. Atlantic halibut in the northwest Atlantic are managed in three Stock Assessment Regions (SAR): the Gulf of Maine and Georges Banks (GM‐GB, blue), the Scotian Shelf and Southern Grand Banks (SS‐SGB, red), and the Gulf of Saint Lawrence (GSL, yellow). The inset map shows the distribution of RV survey abundance counts (1990–2023) across the Hague line (dashed navy), which represents the border between US and Canadian waters. The Exclusive Economic Zone (EEZ, solid navy) demarcates waters where coastal states hold sovereign rights over natural resources, and Northwest Atlantic Fisheries Organization (NAFO) divisions (solid green) are the standardized geographic management areas. Depth contours are at every 100 m up to 500 m and then every 500 m up to 5000 m.

Shifts in species distributions pose a challenge to the management of commercial fisheries, partly because of the common mechanisms used to spatially allocate catch limits across management regions. Typically, fishery removal limits are assigned to a specific area or management unit, in which harvesters are allowed to catch a portion of a stock, often based on the historical distribution across a region. If the distribution of fish among management units deviates from these historical baselines, it complicates the spatial allocation process for assigning catch limits. While distribution shifts can be a challenge within a nation's Exclusive Economic Zone (EEZ, see Figure 1), challenges intensify when the distribution of commercially harvested fish shifts across international borders or between EEZs with differing and independent management and governance structures. This is because without sufficient coordination, independent jurisdictions may fail to detect or respond to cross‐boundary distribution shifts accurately. Globally, 67% of 938 commercial marine stocks are transboundary resources (Palacios‐Abrantes, Reygondeau, et al. 2020). Further, 23% of global transboundary stocks are projected to shift across EEZ borders as early as 2030 (Palacios‐Abrantes et al., 2022).

The potential for economic disruption and conflict between jurisdictions driven by species distribution shifts has led to recommendations for focused research on both governance structure and the science needed to support adaptation (Palacios‐Abrantes et al., 2022; Pinsky et al., 2018; Pinsky, Selden, et al. 2020; Sumaila et al., 2020; Vogel et al., 2023; Woods et al., 2021). In the northern hemisphere, several independent nations conduct their own research surveys to maintain a record of the identity, count, and location of demersal marine species (Maureaud et al., 2021). Commercial stock assessments are typically conducted from these separate surveys for each EEZ and nation. As such, shifts in distribution across national boundaries could be interpreted incorrectly (e.g., a collapse in one jurisdiction is another jurisdiction's emerging fishery). This siloed approach has started to change with increased accessibility to integrated survey databases (Maureaud et al., 2024; Thorson, 2019). Alongside increased data availability, integrated spatiotemporal models have been developed to combine multiple surveys and account for their differences (e.g., in “catchability”), while allowing for a broader, larger geographic perspective (e.g., Thorson, 2019). Such integrated models have been used to understand large‐scale ecological phenomena, such as a region‐wide decline of a coastal shark (Davidson et al., 2025), the large‐scale population structure of a commercial species (Lindegren et al., 2025), and distribution shifts across international borders (O'Leary et al., 2022).

Formerly, broad expectations of global warming were that plants and animals would shift latitudinally towards cooler, more suitable habitats (Parmesan, 2006). Over time, those expectations have been refined (Comte et al., 2020). Across all taxa, 59% of observed range shifts met expectations based on a physiological response to increasing temperatures (Comte et al., 2020; Lawlor et al., 2024). Substantial distribution shifts were expected throughout the Northwest Atlantic based on anticipated warming (Morley et al., 2018), and while there is evidence of marine species' distribution shifts within the United States and Canadian waters, these are not always in the direction anticipated in response to warming, showing that drivers other than temperature can heavily influence species distributions (Fredston et al., 2021; Gordó‐Vilaseca et al., 2023; Kleisner et al., 2016; Lawlor et al., 2024; Mills et al., 2024; Pinsky, Selden, et al. 2020). These varied responses to warming highlight the importance of drawing on ecological knowledge of a species in order to apply the most appropriate approaches to characterize distribution shifts.

Here we use Atlantic halibut (*Hippoglossus hippoglossus*) as a case study to integrate fisheries‐independent surveys that sample juveniles from both the United States and Canada within a spatiotemporal modeling framework. We then use the model output to examine a suite of distribution shift indicators (Link et al., 2021). Atlantic halibut are genetically uniform across the US and Canadian continental shelf, but there are subtle genetic differences between the continental shelf and the Gulf of St. Lawrence (GSL) (Kess et al., 2021). In Canada, Atlantic halibut is managed as two separate stocks: the Scotian Shelf and Southern Grand Banks (SS‐SGB), and the GSL (Figure 1). While Atlantic halibut are distributed across Canadian, US, French territorial, and international waters, this study focuses on the area surrounding the nautical border between Canada and the United States known as the Hague Line (Figure 1). Canadian surveys show that Atlantic halibut abundance has rebounded from an overfished status; biomass and landings have increased over the last 20 years and the fishery was certified as sustainable by the Marine Stewardship Council in 2013 (Johnson et al., 2024; Trzcinski & Bowen, 2016). In contrast, assessments in US waters show that halibut abundance remains low and has been designated as overfished since 2012 and is managed under a rebuilding plan (NOAA, 2024). To support the sustainable and adaptive management of shifting fisheries, this study implements a spatiotemporal modeling approach that integrates US and Canadian fisheries‐independent surveys to model historical spatial distributions and calculates a suite of distribution shift indicators to characterize the expansion of Atlantic halibut. An integrated modeling approach will enable us to focus on a large‐scale research question and provide advice for evidence‐based management.

## MATERIALS AND METHODS

### Data and study area

#### Biological data

Six bottom trawl research vessel (RV) survey data sets, varying in spatial and seasonal coverage, were used in this analysis. (see Appendix S1: Table S1). The Northeast Fisheries Science Center (NEFSC) is a division of NOAA's National Marine Fisheries Service, which conducts spring and fall RV surveys covering an area from Cape Hatteras to the southern part of the Scotian Shelf (Figure 1). Fisheries and Oceans Canada (DFO) Newfoundland conducts spring and fall RV surveys, which extend from the Laurentian Channel and up the coast of Labrador. The DFO Maritimes Region conducts spring and summer RV surveys, covering the Scotian Shelf, the Bay of Fundy and Georges Bank, to the Laurentian Channel. These have the highest proportion of sets with Atlantic halibut presence compared to the other surveys (Appendix S1: Table S1). All surveys use a stratified random sampling design, and there is some spatial overlap between the NOAA NEFSC and DFO Maritimes surveys (Appendix S2: Figure S1). There is fairly consistent coverage across the survey domain from year to year, and the northern range of the Newfoundland surveys (NAFO regions 2GHJ), where coverage is more sporadic, was eventually excluded from the analysis (Appendix S2: Figure S2).

While collectively the surveys range from Cape Hatteras in the United States to the tip of Labrador, Canada, our study area represents the intersection between the footprints of the seasonal NOAA NEFSC, DFO Maritimes, and DFO Newfoundland RV surveys, and the historical Atlantic halibut distribution. Over the last decade, multiple research studies have supported the working theory that Atlantic halibut are spatially structured and exhibit diverse movement patterns. Juveniles, sampled by the RV survey, occur in persistent focal areas across its range (Boudreau et al., 2017). Adult landings are proportional to the size of juvenile habitat (French et al., 2018). Adults can migrate hundreds of kilometers to the slope to spawn in winter and then return to their original areas, exhibiting summer site fidelity; but some stay resident at the summer site year‐round, while a minority of halibut emigrate to other regions (Gatti et al., 2020; Ransier et al., 2024; Shackell et al., 2022).

The Atlantic halibut GSL stock was excluded from this case study, along with NAFO region 3Pn, because GSL fish are frequently present in this area, and its inclusion could interfere with the detection and interpretation of patterns related to the SS‐SGB stock (see Figure 1). We also excluded the extreme southern and northern edges that extend well beyond the observed range of Atlantic halibut (French et al., 2018; Shackell et al., 2016) (see Appendix S1: Table S1) as well as NAFO region 3 M, due to sparse data.

Surveys were originally designed to monitor groundfish abundance, focusing on commercial species. The Atlantic halibut caught in the trawl surveys are predominantly juveniles, as the larger halibut can out‐swim the trawls; the trawl gear has a domed‐shaped selectivity which peaks around 45 cm (Johnson et al., 2024). While the RV surveys catch predominantly juvenile halibut (~50 cm, 1 kg), those of 125 cm (~20 kg) or more have also been caught, which creates high variability in the weight per tow. Disparate RV survey data were combined into a data frame that included details for each unique survey tow (i.e., survey, location, tow, swept area and date) and for the associated biomass and abundance of Atlantic halibut (generating zeros where applicable) within the timeframe of 1990 to 2023.

#### Environmental and historical data

We gathered and processed depth, annual/seasonal average sea surface temperature, and annual/seasonal average bottom temperature data from the Bedford Institute of Oceanography North Atlantic model (BNAM). With a 1/12° resolution, BNAM is an eddy‐resolving North Atlantic Ocean model that was created based on the Nucleus for European Modelling of the Ocean (NEMO) framework to support ocean monitoring (Brickman et al., 2021; Z. Wang et al., 2018). This makes it suitable for this study area, which is often under‐resolved by global ocean climate models (Brickman et al., 2021). Seasonal averages of the BNAM environmental data were calculated annually by grouping monthly values as follows: spring (March–May), summer (June–August), and fall (September–November). RV survey tows were then matched to the BNAM covariate data based on the season and year of collection. To assign environmental values to each tow location, this temporal matching was followed by spatial alignment, using interpolation to average values from the four nearest BNAM grid cells, implemented with the “extract” function from the R *raster* package (Hijmans, 2025).

#### Species distribution model fitting

We fitted Atlantic halibut species distribution models (SDMs) using the vector autoregressive spatiotemporal (VAST) modeling approach, implemented with the R *VAST* package (Thorson, 2019) (Appendix S1: Section S1). Similar to other mixed‐effects SDMs, a VAST SDM can be described as an “environment‐only” SDM enhanced with additional components to account for unexplained temporal, spatial, and spatiotemporal variability in the true underlying abundance as well as sampling differences among observations unrelated to variability in abundance (i.e., catchability covariates) (Thorson, 2019). We fitted abundance (numbers) caught per tow, as this is the standard practice in Atlantic halibut stock assessments (Johnson et al., 2024), with a zero‐inflated Poisson distribution (which involves modeling two linear predictors), a barrier effect (preventing influence from crossing natural boundaries), and anisotropy (to reflect along‐coasts vs. cross‐coast spatial correlation structures). The first linear predictor has a logit‐link and models the proportion of zeros in excess of what is expected from the second linear predictor, which represents the intensity of a log‐linked Poisson distribution. We fitted four alternative VAST models, which all shared this two‐stage structure. All four models used the same approach to account for systematic differences among surveys by including survey (i.e., NEFSC, DFO Maritimes, DFO Newfoundland) as a fixed effect within the catchability component of the model and an offset based on a standard area‐swept (km^2^) for each survey. All models also included season as a fixed effect with three levels (spring, summer, fall), representing differences in expected log density between seasons across space and years.

We first fitted a base (null) intercept‐only model with an AR1 autoregressive structure on annual intercepts for both linear predictors. As the autoregressive parameter (*Rho*_*1*_) approached its upper bound of 1.0, we adjusted the first linear predictor to a random walk process. We used this same intercept autoregressive structure for all subsequent models. Next, we fitted an Environmental Covariates Only model (“Env”), which incorporated the environmental covariates (depth and seasonal average surface and bottom temperatures) as dome‐shaped (quadratic) effects using the bs function in the *splines* R package (Wang & Yan, 2021) with degree equal to two. We then fitted an Environmental Covariates + Spatial model (“Env + Sp”), which included a random effect for persistent spatial variability in encounter probability and positive catch rates. Finally, we fitted a fully specified Environmental Covariates + Spatial + Spatio‐Temporal model (“Env + Sp + SpT”), which added an additional spatiotemporal random effect to account for variability in encounter probability and positive catch rates at each time step. With this full model, we also tried to include an AR1 autoregressive structure on the spatiotemporal components for each linear predictor, though adjusted to a random walk for the first linear predictor following parameter estimation issues. Additional VAST modeling details and equations can be found in Appendix S1: Section S1 and Appendix S1: Table S2.

We fitted models to data from 1990 to 2019, excluding observations from 2019 to 2023 to validate model predictive skill. After fitting each model, we completed general diagnostic checks to confirm model convergence and identify any underlying issues. Convergence checks of residual diagnostics did not show signs of concern with the zero‐inflated Poisson distribution selection (Appendix S2: Figure S3). Moreover, parameter estimate summaries indicated that all models had converged, with final gradients <0.001 (Parameter estimates: Appendix S1: Table S2). We then evaluated fitted models (results in Table 1) by calculating the marginal Akaike information criterion (AIC) to evaluate the model's parsimony (i.e., expected out‐of‐sample predictive performance). Next, we compared each model's prediction skill to the held out 2019–2023 observations. We calculated the area under the receiver‐operator curve (AUC) using the AUC function in the *MLmetrics* R package (Yan, 2024) as a measure of presence/absence classification ability, and we quantified prediction accuracy using root mean squared error (RMSE) and mean absolute error (MAE) with the accuracy function in the *MLmetrics* R package (Yan, 2024).

**TABLE 1:** Model fit and predictive performance for four alternative species distribution models.

Model	Marginal AIC	Delta AIC	AUC	RMSE	MAE	*n*. fixed effects
Env + Spy + SpT	24346.60	0	0.88	0.56	0.15	32
Env + Sp	24793.84	447.24	0.87	0.63	0.17	29
Env	29035.90	4689.3	0.79	0.69	0.21	25
Null (intercept only)	31481.99	7135.39	0.69	0.70	0.21	5

*Note*: Includes a null (intercept only) model, a model with environmental covariates (“Env”), a model with environmental covariates and spatial random effect (“Env + Sp”), and a fully specified model with environmental covariates, spatial, and spatio‐temporal random effects (“Env + Sp + SpT”). Increasing model complexity is also reflected with the number (*n*.) of fixed effects that were included in each model. Marginal and delta AIC were used to evaluate the model's parsimony, and predictive performance statistics (AUC, RMSE, and MAE) were all calculated by comparing model predictions to excluded observations between 2019 and 2023.

Abbreviations: AIC, Akaike information criterion; AUC, area under the receiver‐operator curve; MAE, mean absolute error; RMSE, root mean squared error.

#### Shift indicators

Animal distributions respond to a variety of ecosystem conditions, and it is important for fisheries management to identify those changes in productivity and distribution driven by climate change (Link et al., 2021). To quantify and understand Atlantic halibut distribution and abundance changes, we selected the best‐fitting and predictive SDM (Env + Sp + SpT) and then used it with the BNAM gridded environmental data to make continuous year‐season predictions across the study domain from 1990 to 2023. Since spring had the best survey coverage, with sampling in all three regions (Appendix S1: Table S1), we isolated yearly spring model estimates for our shift indicator analysis.

Following Link et al. (2021)'s proposed business rules, we used the model predictions to calculate a suite of six indicators of shift and expansion to identify, assess, and categorize the nature of spatial or temporal changes in abundance across the study region: Abundance, Centre of Gravity (COG), Abundance‐weighted Depth (AWD), Area Occupied (AO), Leading and Trailing Range Edge, and Distance to Border (i.e., distance from COG to the international border at the Hague line). COG was calculated using centroid formulas that calculate the average latitude and longitude, weighted by abundance. AWD was calculated as the average depth, weighted by abundance. AO was calculated using an index representing the area containing 90% of the abundance. Leading and Trailing Range Edges were calculated based on the geographical minimum (5%) and maximum (95%) of modeled abundance. Distance to Border was calculated using geospatial tools (e.g., Haversine formula) to compute the shortest path from the COG to the nearest point on the international border (the Hague line), implementing a rule to ensure that this path did not cross land.

We calculated each indicator as a time series within two distinct warming periods, at a regional scale (United States and Canada), and then within focal areas (Appendix S1: Table S3). Warming waters have already led to changes in species productivity in the Gulf of Maine region (Pershing et al., 2015) and temperatures are projected to continue to rise (Brickman et al., 2021). Across the study domain, an increase in the frequency of naturally occurring warm water influxes resulted in an enhanced warming trend that started around 2005–2007 (Brickman et al., 2018; Friedland et al., 2024) leading to a thermal regime shift by 2012 in the Gulf of Maine region (Friedland et al., 2024). The increase in Atlantic halibut abundance in Canada (starting around 2005) has been associated with an increase in thermally desirable habitat available and an increase in growing degree days across the region (Czich et al., 2023). In this analysis, we evaluated and compared temporal trends in abundance and shift indicators using a linear model in two periods: before (1990–2005) and during (2006–2023) enhanced warming. We assumed a Gaussian error distribution and extracted the slopes and confidence intervals to compare rates between the two periods.

**FIGURE 2:**
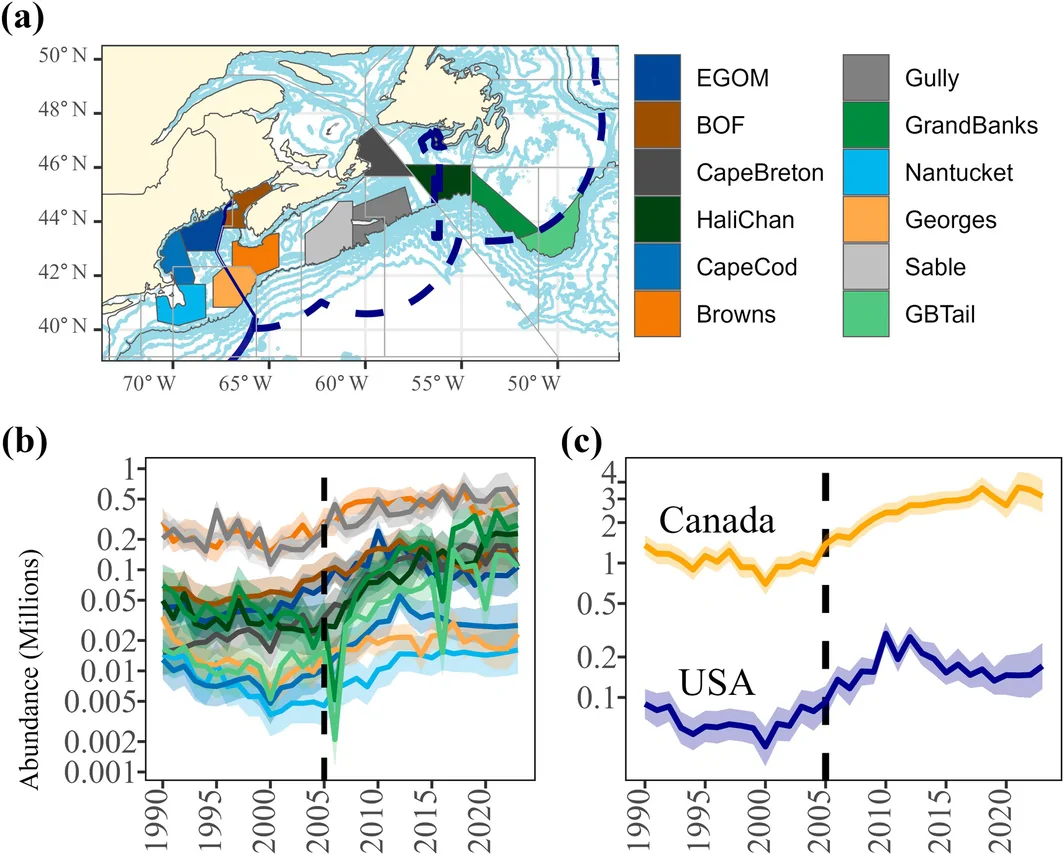
Focal Areas and estimated abundance trends. (a) Focal Areas: Eastern Gulf of Maine (EGOM), Bay of Fundy (BOF), Cape Breton (CapeBreton), Halibut Channel (HaliChan), Cape Cod (CapeCod), Browns Bank (Browns), Gully (Gully), Grand Banks (GrandBanks), Nantucket Shoals (Nantucket), Georges Bank (Georges), Sable Island Bank (Sable), and the Tail of the Grand Banks (GBTail), (b) Annual modeled abundance (millions) and confidence intervals for Atlantic halibut (*Hippoglossus hippoglossus*), with vertical axis on a log scale, in all focal areas, and in (c) Canada and US regions. The dashed vertical line is at 2005, dividing the time series into 1990–2005 and 2006–2023. Canada accounted for 94% of the total abundance in both periods.

To capture the population structure and to support fine‐scale analyses of this broadly distributed species, we further divided the study area into focal areas designed to represent the spatial stock structure of Atlantic halibut. The use of focal areas enabled the application of the shift indicators to a spatially structured population. Twelve focal areas were identified within the study region based on five sources of evidence relating to Atlantic halibut stock structure and distributions (Figure 2a) (Boudreau et al., 2017; Kersula & Seitz, 2019; Liu et al., 2019; Ransier et al., 2024; Seitz et al., 2016; Shackell et al., 2022). To ensure that the size of the focal areas was not too restrictive, we conducted several iterations using different sized polygons. For the first iteration, we created polygons around identified focal areas, and then estimated the 0.05, 0.5, and 0.95 abundance quantiles for each year within each. If the 0.05 or 0.95 quantile indices were at a maximum and/or showed no variation over the time series, we increased the polygon size for the next iteration until there was at least some variation in the 0.05 and 0.95 quantiles. Descriptions of the focal areas are found in Appendix S1: Table S3.

## RESULTS

### Species distribution model evaluation

The three fitted Atlantic halibut SDMs (Env, Env + Sp, and Env + Sp + SpT) each estimated similar spatial and temporal trends in Atlantic halibut abundance. Overall, there appeared to be important spatial and spatiotemporal patterns in the data that could not be explained solely by the environmental covariates, contributing to a progressive increase in model performance with the addition of spatial and then spatiotemporal random effects. The fully specified Env + Sp + SpT model had the best fit and was therefore used for the development of the subsequent portion of this analysis (Table 1). It offered the best balance between model fit and complexity (lowest marginal AIC) and was the best predictive model with the highest AUC, indicating a strong ability to distinguish between positive and negative outcomes, and the lowest RMSE and mean absolute error (MAE) (Table 1). Each season showed similar spatial and temporal estimates, patterns and trends, further supporting the isolation of spring estimates for the calculation of shift indicators (Appendix S2: Figure S4).

### Regional and focal area abundance trends

**FIGURE 3:**
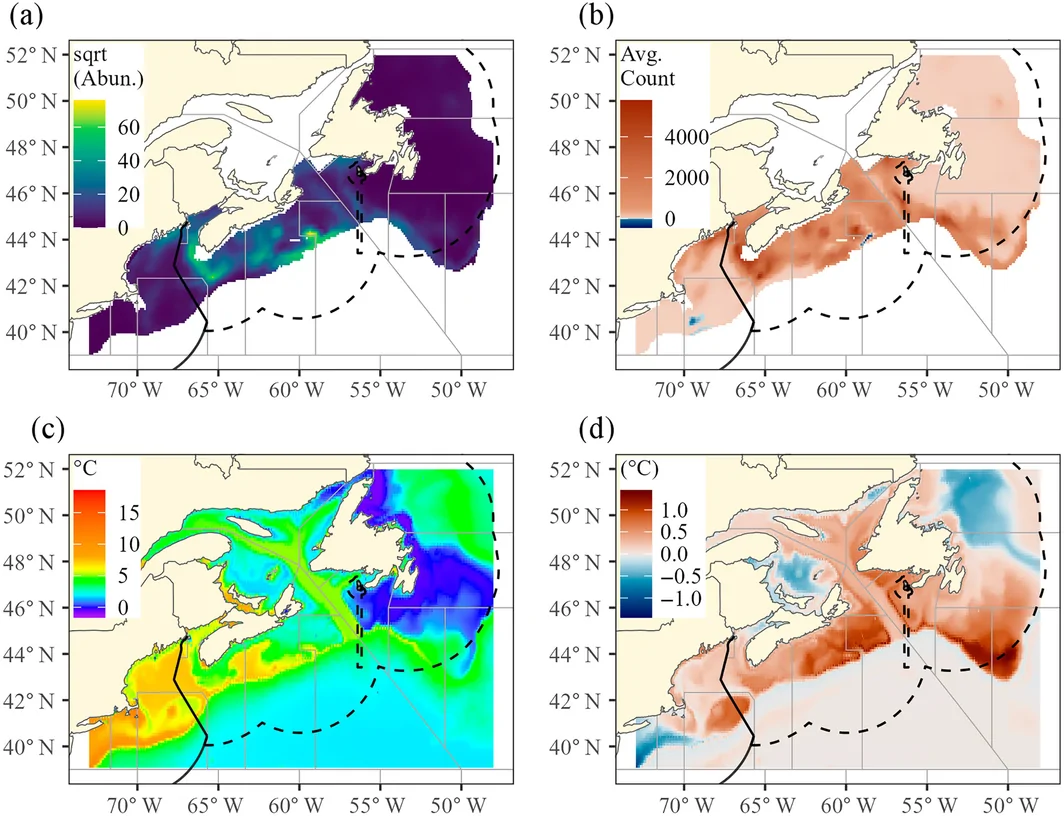
Spatial estimates of Atlantic halibut abundance, and bottom temperature. Estimated spatial abundance of Atlantic halibut (*Hippoglossus hippoglossus*) and bottom temperature (1990–2005) and the difference gained/lost in the 2006–2023 period. (a) Mean estimated abundance for 1990–2005; here, abundance estimates were square‐root‐transformed (sqrt Abun.) and interpolated using bivariate linear interpolation for mapping purposes. (b) Gain or loss in average (Avg.) abundance count: Absolute difference in the mean estimated abundance for 2006–2023 less the abundance for 1990–2005, with gain scaled in red and loss scaled in blue. (c) Mean annual bottom temperature 1990–2005 in degrees Celsius (Bedford Institute of Oceanography North Atlantic model [BNAM]). (d) Warming or Cooling signal: Absolute difference in mean annual bottom temperature in the 2006–2023 period less the 1990–2005 period (degrees Celsius), with warming scaled in red, and cooling scaled in blue.

Abundance increased in all focal areas (Figure 2a) and in both the United States and Canada during the 2006–2023 period (Figure 2b,c). Throughout the time period, Atlantic halibut in Canadian waters have accounted for 94% of their total abundance in the study area. At the regional level, the rate of change in abundance was significantly positive in Canada during the 2006–2023 period but not in the United States, where the increase had leveled off around 2012 (Appendix S2: Figure S5). Across the entire study region, abundance increased during the 2006–2023 period, especially in the northeast, where the greatest increase in temperature also occurred (Figure 3). This is reflected in the rates of change among focal areas where the rates of change in abundance were significantly positive and higher across the northeastern part of the range, as well as in the most southerly focal area (Appendix S2: Figure S5).

### Centre of gravity and abundance‐weighted depth

**FIGURE 4:**
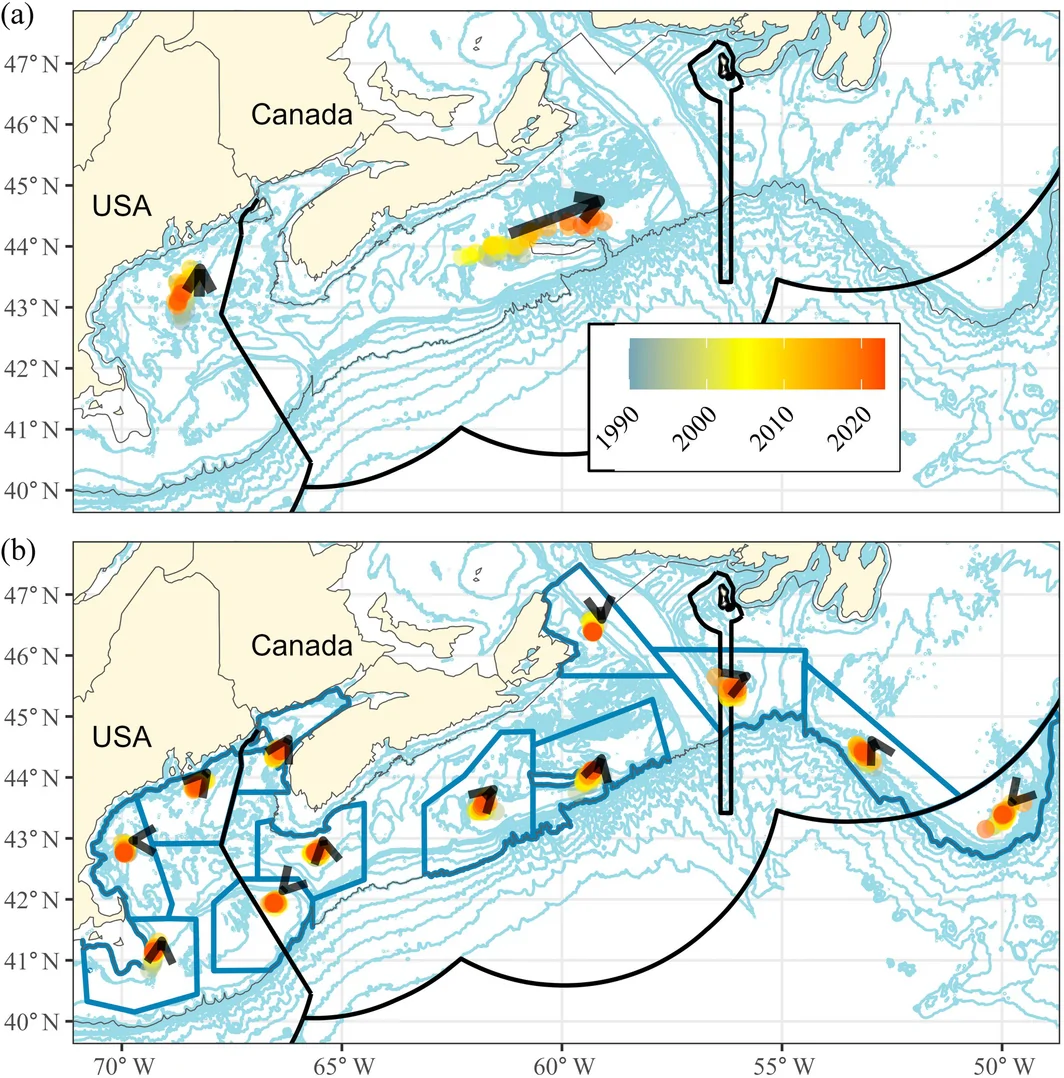
Annual Centre of Gravity (COG) of Atlantic halibut (*Hippoglossus hippoglossus*) from 1990 to 2023. COG representing the average latitude and longitude, weighted by modeled abundance estimates in: (a) Canada and the United States and (b) each focal area. Color gradient represents the annual COG (year). The arrow start represents the geographical position at the start of the time series, and the arrow head represents the direction and end of the time series. Arrows are slightly offset for clarity.

The net direction of movement of the annual COG was northeast in Canada over the full time period; it shifted in both periods of 1990–2005 and 2006–2023 at a rate of 3 and 12 km per year, respectively (Figure 4a, Appendix S1: Table S4). The net direction of movement of the annual COG was north in the United States. It shifted in earlier and later periods at a rate of 5 and 2 km per year, respectively (Figure 4a, Appendix S1: Table S4). The shift in Canadian waters is consistent with the continental shelf topography of the region, which lies on a northeast axis, while the shift to the north in the United States represented a shift towards the coast.

We examined patterns at the level of focal areas in consideration of Atlantic halibut spatial structure (Boudreau et al., 2017). We had created the focal areas to be similar in size and large enough to potentially detect distribution shifts (see methods), but acknowledge their size may still limit shift detection, so here we focus on overall net direction. Our expectation, given warming and/or a population increase, is that the COG of focal areas would shift northeastward across most areas. However, the movement patterns of COG varied among focal areas and time periods; this variability did not conform to our original expectations (Figure 4b; Appendix S2: Figure S6). The COG of Nantucket Shoals, Bay of Fundy, Browns Bank, and Gully shifted slightly north or northeast, while Cape Breton shifted in a southerly direction and Grand Banks shifted northwest (Appendix S2: Figure S6; Appendix S2: Figure S7). Across all focal areas, the net distance of COG shifts ranged from 7 to 29 km (Appendix S1: Table S4); nonetheless, we focused on the net directional patterns as net distance may be limited by the spatial extent of the focal area polygons.

In some cases of demersal fish species, a response to warming can include a shift to deeper waters (Dulvy et al., 2008; Freitas et al., 2021). We examined the AWD at the regional level and observed no significant changes in depth in either region (Appendix S2: Figure S8). However, at the scale of focal areas, the AWD has become shallower in all focal areas west of Sable Island Bank (Appendix S2: Figure S9).

### Leading and trailing range edge and area occupied

**FIGURE 5:**
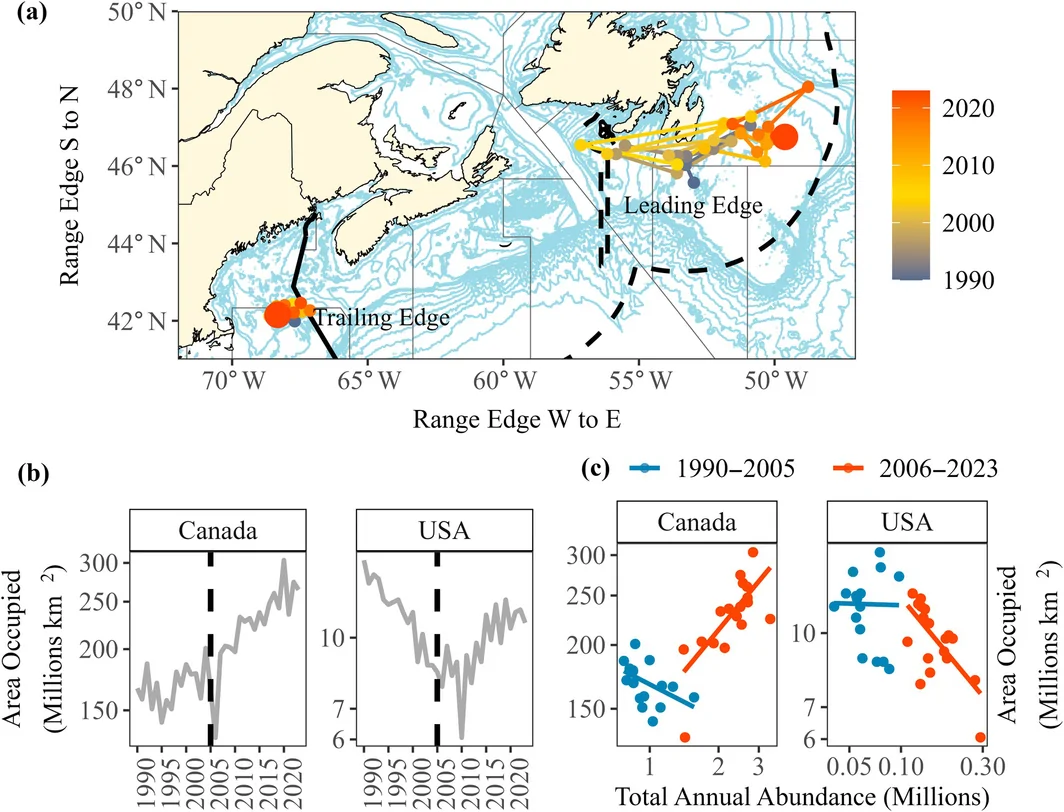
Shift indicators: Leading and trailing range edge, area occupied and relation with abundance. (a) Mapped Leading and Trailing Range Edge of distribution of modeled Atlantic halibut (*Hippoglossus hippoglossus*) abundance estimates from 1990 to 2023. Expansion would occur in the northeast (NE) or southwest (SW) directions consistent with the topography of the study domain. The terminal year, 2023, is displayed as a larger dot. (b) Area Occupied (AO) in Canada and the United States from 1990 to 2023, and (c) relationship between AO and total annual abundance during 1990–2005 and during 2006–2023 in both regions.

In 1990–2005, the Leading Range Edge shifted 43 km north and 51 km west, a total distance of ~66 km. From 2006 to 2023, the Leading Range Edge shifted 62 km north and ~ 264 km east, resulting in a total shift of 271 km northeast. From 1990 to 2005, the Trailing Range Edge shifted 18 km north and 4 km east for a total shift of ~20 km. From 2006 to 2023, the Trailing Range Edge shifted 12 km north and 69 km east, resulting in a total shift of ~70 km northeast (Figure 5a). The results indicate a predominantly eastward expansion on the Leading Range Edge along the northeast axis of the continental shelf.

During 1990–2005, the AO was stable in Canada and decreased in the United States (Figure 5b). During 2006–2023, the AO increased over time for each region, Canada and the United States (Figure 5b). The relationship between AO and total abundance was not significant in either of the regions during the 1990–2005 period (Figure 5c). During 2006–2023, the relationship between AO and total abundance was significantly positive in Canada, indicating a spatial expansion as abundance increased (Figure 5c). The relationship between AO and total abundance was significantly negative in the United States during the 2006–2023 period (Figure 5c), when abundance was higher (Figure 2c), suggesting that density or number per unit area is decreasing within the United States.

### Distance to international border

**FIGURE 6:**
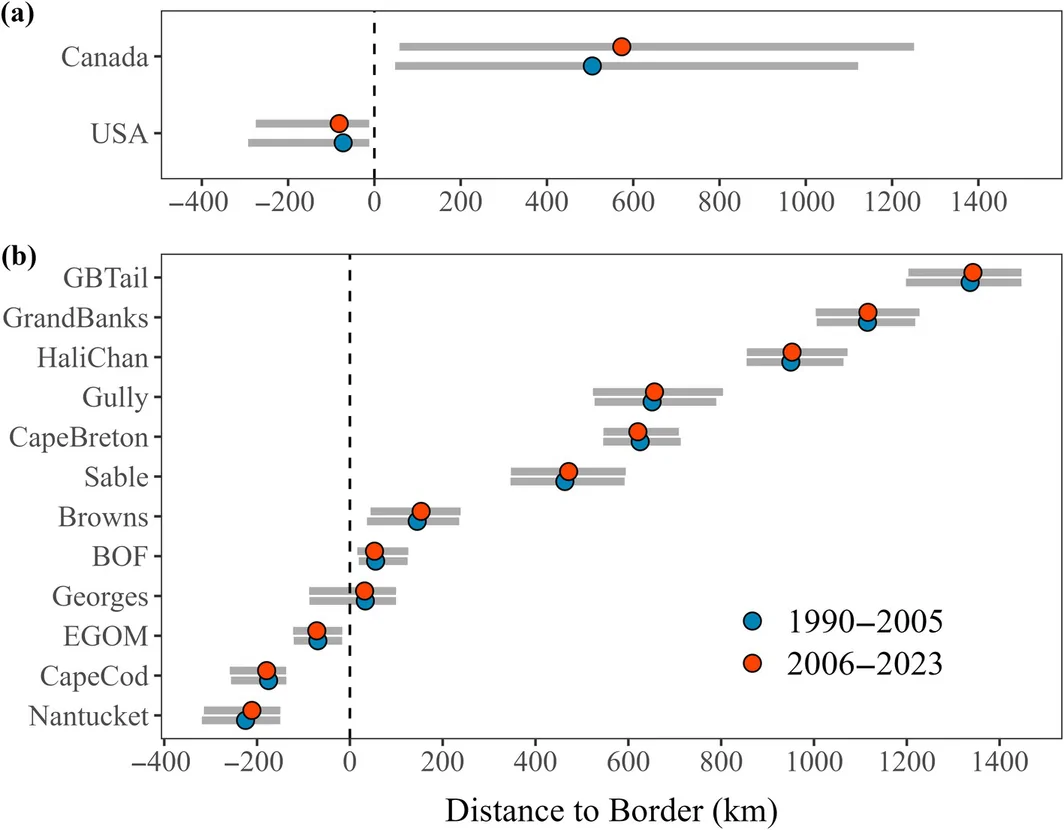
Distance to International Border (Hague line). The international border is represented by the dashed line at 0, with negative values representing westward and positive values representing eastward movement. Values are the 5th, mean, and 95th quantile of the density of Atlantic halibut distribution during 1990–2005 (blue) and 2006–2023 (red), for (a) Canada and United States and (b) focal areas, arranged roughly north to south: Tail of the Grand Banks (GBTail), Grand Banks (GrandBanks), Halibut Channel (HaliChan), Cape Breton (CapeBreton), Gully (Gully), Sable Island Bank (Sable), Browns Bank (Browns), Bay of Fundy (BOF), Georges Bank (Georges), Eastern Gulf of Maine (EGOM), Cape Cod (CapeCod), and Nantucket (Nantucket Shoals).

The distances from the COG to the jurisdictional border (Hague line) have not changed appreciably during either period in both regions, nor in any of the focal areas (Figure 6 a, b). Georges Bank spans the Hague Line, and the 5th and 95th quantiles of focal areas adjacent to the international border (especially Eastern Gulf of Maine and Bay of Fundy) reflect a potential for mixing across the border (Figure 6b).

### Summary: Interpreting the suite of shift indicators

**FIGURE 7:**
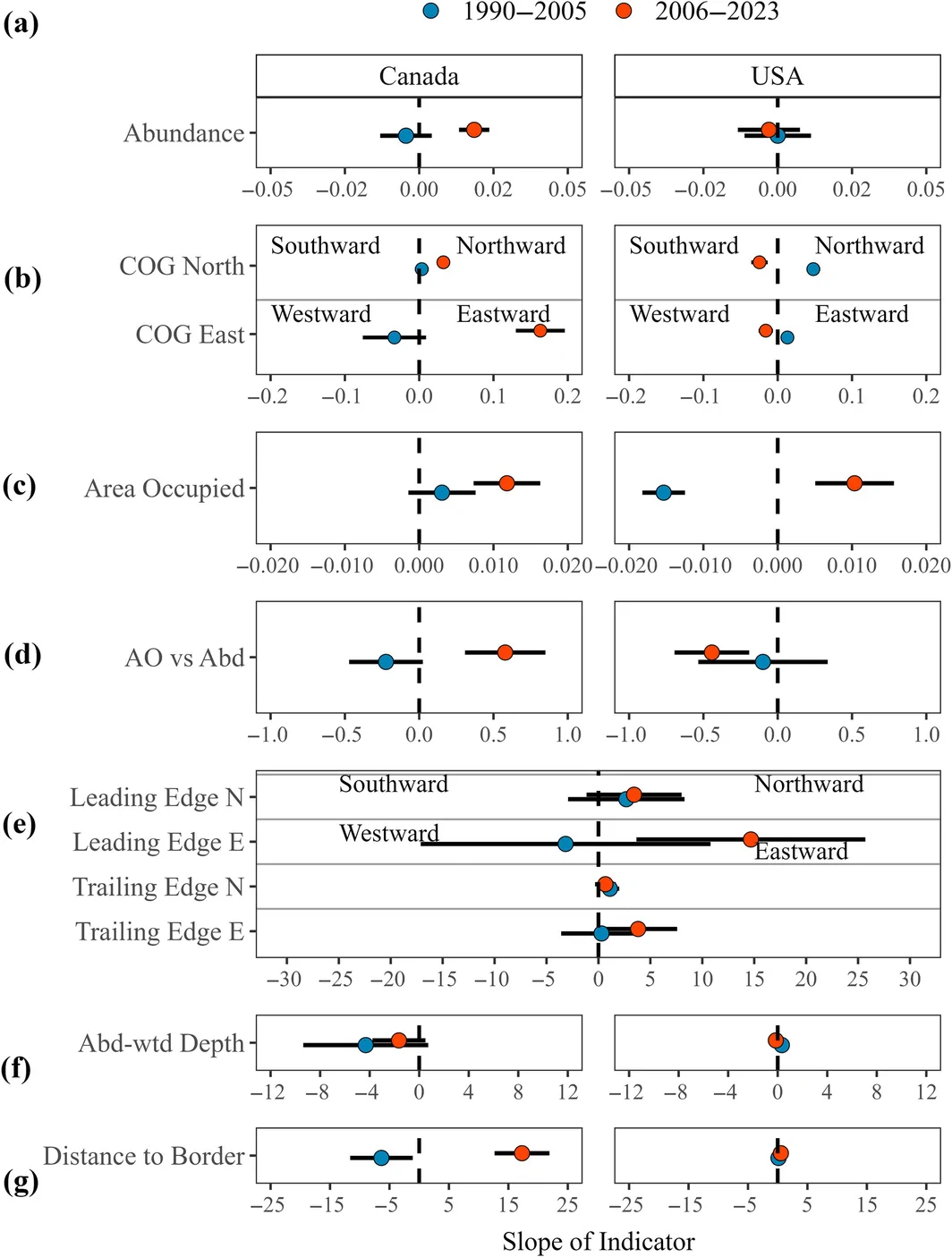
Regional Summary of Shift Indicator Slopes. Values are the slope and confidence interval (CI) for Atlantic halibut (*Hippoglossus hippoglossus*) abundance over time within two periods, 1990–2005 (blue) and 2006–2023 (red), for Canada (left) and United States (right). All slopes are centered between positive and negative values in original units (0 represented with a dashed line). (a) Abundance. (b) Centre of Gravity (COG) for both latitudinal (North) and longitudinal (East) shifts: positive slopes represent northward and eastward, and negative slopes represent southward and westward movement. (c) Area Occupied (AO). (d) AO versus Abundance (Abd), (e) Leading and Trailing Range Edge for both latitudinal (N) and longitudinal (E) shifts: positive slopes represent northward and eastward, and negative slopes represent southward and westward movement. (f) Abundance‐weighted (Abd‐wtd) Depth: positive slopes represent deepening and negative slopes represent shallowing. (g) Distance to Border: positive slopes represent movement away and negative slopes represent movement towards the international border (Hague line).

Following Link et al. (2021), we interpreted the suite of shift indicators by comparing their rates of change across regions and focal areas, largely focusing on the 2006–2023 period. Firstly, abundance increased in Canada and in the United States from the early 2000s but then leveled in the United States (Figure 2c) while it continued to increase in Canada at a rate of 4.4% in terms of number/year (95% CI: 3.1–5.6%) from 2006 to 2023 (Figure 7a). During the 2006–2023 period in Canada, the COG shifted northeast at a rate of 4 km/year north (95% CI: 3–4.3 km) and 12 km/year east (95% CI: 10–15 km) (Figure 7b). During the same period in the United States, the COG shifted southwest at 3 km/year south (95% CI: 1.5–4 km) and 1 km/year west (95% CI: 0.5–2 km) (Figure 7b). From 2006 to 2023, AO in both Canada and the United States increased at a rate of 2.8% km/year (95% CI: 1.7%–3.8%) and 2.4% km/year respectively (95% CI: 1.2%–3.7%) (Figure 7c). From 2006 to 2023, AO increased by 0.58% for every 1% increase in abundance in Canada (Figure 7d). In contrast, over the same period, AO decreased by 0.44% for every 1% increase in abundance in the United States (Figure 7d).

From 2006 to 2023, the Leading Range Edge shifted 3.4 km north per year (95% CI: 2.92 to 8.29 km) and 14.68 km east per year (95% CI: 3.66 to 25.7 km). The Trailing Range Edge shifted at a much slower rate of 0.67 km north per year (95% CI: 0.33 to 1.67 km) and 3.82 km east per year (95% CI: 0.05 to 7.58 km) (Figure 7e). There was no significant evidence of deepening in Canada nor the United States from 2006 to 2023 (Figure 7f). From 2006 to 2023, in Canada, the distance from the population centroid to the Hague Line (Distance to Border) increased at a rate of 17.3 km/year (95% CI: 12.68 to 21.92 km) and by 0.53 km/year (95% CI: 0.09 to 1.15 km) in the United States (Figure 7g). In conclusion, juvenile Atlantic halibut have increased in abundance, occupying more area, and expanded northeast during the warmer 2006–2023 period, relative to the cooler 1990–2005 period.

## DISCUSSION

We have provided evidence of a range expansion in a spatially structured stock that grew rapidly as waters warmed (Czich et al., 2023) after recovering from overfishing (Johnson et al., 2024; Trzcinski & Bowen, 2016). Overall, the population is expanding in a northeasterly direction, but there is no evidence of consistent directional shifts within focal areas. The northeast shift and expansion are consistent with the shelf's topography, which also follows a northeast axis, and with a concomitant increase in both abundance and area occupied. Interpreting the suite of indicators together in a comprehensive large‐scale assessment is important because they collectively tell the story of shifts and range expansion (Link et al., 2021).

### Transboundary science advice to inform fishery management

The ability to calculate spatial indicators is made possible by the availability of long‐term monitoring data in both Canada and the United States (Shackell et al., 2016) and can be used to inform bilateral discussions in support of the effective management of this transboundary species. Consider, in this study, the proximity of the southerly focal areas (Eastern Gulf of Maine, Bay of Fundy, Georges Bank, and Browns Bank) to the international border. Observations of electronically tagged fish suggest residency within the Gulf of Maine (Kersula & Seitz, 2019; Seitz et al., 2016), but that mixing can occur during seasonal spawning migrations to the slope (Liu et al., 2019). Conventional tagging showed that 28% of fish tagged in the Eastern Gulf of Maine have been caught in adjacent waters on the Canadian side (Kanwit, 2007). The indicator of Distance to Border over time represents a useful and simple indicator to monitor shifts towards or away from the shared border. Furthermore, marine species range edges typically maintain their thermal niche, although there are exceptions (Fredston et al., 2021). Often, the colder edge will move faster; that is, range expansion can occur before range contraction (Fredston‐Hermann et al., 2020). Consistent with the axis of the study domain, as well as the expectations outlined by Fredston‐Hermann et al. (2020), we detected a northeast shift in the Leading Range Edge during the warmer 2006–2023 period which is suggestive of a response to temperature change.

The assessment and fishery management of Atlantic halibut does not have a formalized transboundary process, but there is growing bilateral interest in Atlantic halibut due to its high economic value and the recognition that there is a propensity for these fish to cross the Hague line (Shackell et al., 2022). Examples of the successful provision of transboundary science advice exist between Canada and the United States with regard to Pacific halibut and Pacific hake (IPHC, 2021; Johnson et al., 2025; Webster et al., 2020). In each case, species abundance data from both the United States and Canada are incorporated into assessment models, and this science advice is provided to relevant management bodies on either side of the border. Here, by incorporating spatially resolved environmental data to indicate how and where the species is moving within and between international borders, we provide a collaboratively produced (United States and Canada) SDM that provides insights that are not possible using traditional stock assessment methods. The work can be used as additional information to support and confirm traditional model outputs, or incorporated directly into the stock assessment (McDonald et al., 2021). This advancement builds on both countries' efforts to improve ecosystem approaches to fisheries management and adopt tools that allow for adaptive management.

Globally, the ability to assess distribution shifts is improving (Grüss et al., 2025) but is still constrained by limited sharing of data collected independently by neighboring jurisdictions, as well as a need for a clear methodology to test whether shifts are driven by climate change (Maureaud et al., 2021, 2024; Taheri et al., 2021). Here, we have provided an approach that uses the output from a spatiotemporal model to estimate distribution shift indicators, which are then interpreted as an ensemble (Link et al., 2021). However, the operational linkage between the science output and fisheries management is weak. There is a general need for fisheries‐focused decision‐support frameworks that would enable science advice regarding distribution shifts to inform decision‐making across multiple management areas. Such frameworks would facilitate an ongoing dialogue between science and stakeholders, in order to create, translate, and communicate scientific advice in a management‐relevant and actionable form (DePiper et al., 2017; Levin et al., 2009). Integrated spatiotemporal models and a suite‐interpretation of spatial shift indicators, as in this case study, demonstrate the foundation necessary for the development of such a framework. The framework could also include climate adaptation strategies that may be relevant at regional and stock scales, including spatially re‐allocating catch limits, limiting or enhancing fisheries, and opening or closing fishing areas (e.g., Free et al., 2025; Woods et al., 2021). While implementation would require collaborations among jurisdictions within existing regulatory settings, operationalizing distribution shift indicators could address globally relevant challenges: sustainable management of harvested marine species and mitigation of economic impacts from climate‐driven changes. (Palacios‐Abrantes, Sumaila et al., 2020; Pinsky, Rogers, et al. 2020; Vogel et al., 2023).

## CONFLICT OF INTEREST STATEMENT

The authors declare no conflicts of interest.

## Supporting information


Appendix S1.

Appendix S2.

## Data Availability

Data and code (Ferguson et al., 2026) are available in Zenodo at https://doi.org/10.5281/zenodo.19005016.
