# Supplementary material for: Shifts and expansion in a spatially structured fish stock spanning international borders in the Northwest Atlantic
Source: Ecol Appl. 2026 Sep 16;36(6):e70311. doi: 10.1002/eap.70311 (PMC13581450; doi:10.1002/eap.70311)
Supplement: Supplementary file 1 [file EAP-36-e70311-s001.pdf]

Appendix S1

Shifts and expansion in a spatially structured fish stock spanning international borders in the Northwest Atlantic

Nancy L Shackell, Kiyomi J Ferguson, Andrew J. Allyn, Cornelia E. den Heyer, Jamie C. Tam, David M Keith, Katherine E. Mills, James T. Thorson

Ecological Applications

Table S1: Details of research vessel (RV) surveys.

Agency/ Survey/ Region	Season	Depth (m)	Swept (NM ²)	n	% with P	Vessels/ Gear Type/ Tow Protocol	Filters
NOAA NEFSC, bottom trawl, Northeast USA Continental Shelf^ (Miller et al., 2010)	Spring	~18 - 400	0.0384	7791	2.9	Albatross IV pre-2008: Yankee 36 (30 mins- 3.0kts), Henry B. Bigelow >2008: 4-seam/3-bridle bottom trawl with rockhopper sweep (20 mins- 3.0 kts)^^^	<73.34° West, 1990-2023
NOAA NEFSC, bottom trawl, Northeast U.S. Continental Shelf^ (Miller et al., 2010)	Fall	~18 - 400	0.0384	7755	2.2	Same as NEFSC Spring	<73.34° West, 1990-2023
DFO MAR bottom trawl, NAFO divs: 4VWX (Claytor et al., 2014)	Spring	~25 - 750	0.0405	5334	11.3	Alfred Needler: Western IIA trawl (30-min tow at 3.5 kts)	1990-2023
DFO MAR, bottom trawl, NAFO divs: 4VWX5Y (Claytor et al., 2014)	Summer	~25 - 750	0.0405	7682+ 145^^	19.5	Same as DFO MAR Spring	1990-2023
DFO NF region, bottom trawl, NAFO divs: 3LNOP (DFO, 2024)	Spring	~30 - 750	Wing spread * tow dist.	11933 + 4812^^	4.7	Wilfred Templeman/ Alfred Needler & Teleost. 1995: Moved from Engel to Campelen (15-min tow at 3.0 kts)^^^	NAFO: 3KLNOPs 4Vns, 1990-2023
DFO NF region, bottom trawl, NAFO divs: 2HJ, 3KLNO (DFO, 2024)	Fall	~30 - 1500	Wing spread * tow dist.	13031 + 1633^^	2.5	Same as DFO NF Spring	NAFO: 3KLNOPs 4Vs, 1990-2023

^ Coastal and offshore waters from Cape Hatteras, North Carolina to the Gulf of Maine, including Georges Bank

^^ DFO Maritimes summer samples taken during fall months were classified as fall, and Newfoundland spring/ summer samples taken during summer months, were classified as summer for the analysis

^^^Data have been adjusted for changes in vessel/gear

All surveys have a stratified random sampling design, details from (Miller et al. 2010; Claytor et al. 2014; DFO 2024)

Abbreviations: Number (n.), presence (P), the National Oceanic and Atmospheric Administration (NOAA), the Northeast Fisheries Science Center (NEFSC), the Department of Fisheries and Oceans Canada (DFO), Maritimes (MAR), North Atlantic Fisheries Organization divisions (NAFO divs), Newfoundland (NF).

Section S1: VAST Modeling Details

Spatio-temporal model structure

We fit spatio-temporal models to Atlantic halibut (*Hippoglossus hippoglossus)* abundance data as zero-inflated Poisson delta models, consisting of two linear predictors (i.e., stages), with the first stage (n) modeling the zero-inflation component of the Poisson distribution with a logit link (S1.1) and the second stage (w) modeling the mean intensity of the log-linked Poisson distribution (S1.2)(Thorson 2019). All four models shared a similar basic structure, which included autoregressive processes on year-season temporal intercepts, fixed effects for survey within the catchability component of the model with three levels (NEFSC, DFO Maritimes, DFO Newfoundland), and finally, season as a fixed effect with three levels (spring, summer and fall) within the habitat density component. Each of the environmental covariates ("Env"), environmental covariates + spatial ("Env + Sp") and environmental covariates + spatial + spatio-temporal ("Env + Sp + SpT") models then represented alternative configurations through the addition of spatial or spatio-temporal random effects. The model formulas for (n) and (w) are then expressed as a function of temporal intercepts β, spatial variation ω, spatio-temporal variation ε, density covariates X, and catchability covariates Y:

$$logit\left(n(s_i,t_i)\right) = \beta_n(t_i) + \omega_n(s_i) + \varepsilon_n(s_i,t_i) + \sum_{p1=1}^{n_{p1}} \left(\gamma_n(t_i,p1)\right) X_n(i,t_i,p1) + \sum_{k1=1}^{n_{k1}} \lambda_n(k1) Y_n(i,k1) \tag{S1}$$

$$log\left(w(s_i,t_i)\right) = \beta_w(t_i) + \omega_w(s_i) + \varepsilon_w(s_i,t_i) + \sum_{p2=1}^{n_{p2}} \left(\gamma_w(t_i,p2)\right) X_w(i,t_i,p2) + \sum_{k2=1}^{n_{k2}} \lambda_w(k2) Y_w(i,k2) \tag{S2}$$

Density covariates X_n and X_w represent drivers of fish abundance, including season, depth, annual seasonal average bottom and sea surface temperature. In contrast to the density

covariates, catchability covariates λ_n and λ_w are nuisance variables (e.g., monitoring program factors), which account for sampling differences among observations rather than variation in true underlying fish abundance (Maureaud et al. 2021). Catchability covariates can be estimated as fixed or random effects, where $k1$ indexes catchability covariates in the first linear predictor; n_{k1} is the number of catchability covariates in the first linear predictor and $\lambda_n(k1)$ is the effect of catchability covariate $k1$ for the first linear predictor. All parameters for the second linear predictor $p2$, n_{p2}, $\gamma_w(t_i, p2)$, $k1$, n_{k2}, and $\lambda_w(k2)$ have similar meanings.

The spatial variation terms ω_n and ω_w and the spatio-temporal variation terms ε_n and ε_w account for unmeasured variables and processes that can create variation in abundance not explained by density covariates (Thorson et al. 2015). Spatial variation is estimated for the entire study period, while spatio-temporal variation is estimated for each individual time step (i.e., study year-season). Both the spatial and spatio-temporal terms are random effects and are modelled as Gaussian Markov Random Fields (GMRFs) following a multivariate normal (MVN) distribution (Thorson et al., 2015). The spatial structure of the spatial and spatio-temporal variation terms is governed by the covariance matrix of the GMRFs, which specifies the correlation between any two locations based on their distance and direction apart (Thorson et al., 2015). Given the cost of calculating GMRF values for every unique sample location (Rue and Held 2005), *VAST* uses the stochastic partial difference equation (SPDE) to approximate the GMRFs over a constant spatial mesh created with the *R-INLA* R package (Rue et al. 2009; Lindgren et al. 2011). The spatial mesh is defined by (i) the number of knots, which set the grain or resolution of the spatio-temporal model and (ii) the knot locations. We specified 400 knot locations and then relied on the internal k-means clustering algorithm to place these knots within the study domain. With the created spatial mesh, the correlation values between knot locations can be estimated using the SPDE to approximate

the Matérn autocorrelation function developed in Thorson et al. (2015) that accounts for geometric anisotropy (their Equation (4)).

Although temporal variation terms (β_n and β_w) and spatio-temporal variation terms (ε_n and ε_w) are typically treated as fixed effects, they can instead be modelled as random effects with an auto-regressive structure. This allows for sharing information among season-years (Thorson 2019; Charsley et al. 2023) and can be represented as:

$$\beta_n(t) \sim \{ Normal(0, \sigma^2_{\beta_n}) \; if \; t = t_{min} \; Normal(\rho_{\beta_n}\beta_n(t-1), \sigma^2_{\beta_n}) \; if \; t > t_{min} \} \quad \text{(S3)}$$
$$\beta_w(t) \sim \{ Normal(0, \sigma^2_{\beta_w}) \; if \; t = t_{min} \; Normal(\rho_{\beta_w}\beta_w(t-1), \sigma^2_{\beta_w}) \; if \; t > t_{min} \}$$

where t_{min} is the first modelled year; ρ_{β_n} and ρ_{β_w} are the estimated degree of autocorrelation in temporal variation; and $\sigma^2_{\beta_n}$ and $\sigma^2_{\beta_w}$ are the variance of temporal variation ρ_{β_n} and ρ_{β_w}. Similar to the autoregressive temporal variation structure, an autoregressive structure to the spatio-temporal variation terms can be incorporated as:

$$\varepsilon_n(t) \sim \left\{ MVN\left(0, \sigma^2_{\varepsilon_n} R(\kappa_n)\right) \; if \; t = t_{min} \; MVN\left(\rho_{\varepsilon_n}\varepsilon_n(t-1), \sigma^2_{\varepsilon_n} R(\kappa_n)\right) \; if \; t > t_{min} \right\}$$

$$\varepsilon_w(t) \sim \left\{ MVN\left(0, \sigma^2_{\varepsilon_w} R(\kappa_w)\right) \; if \; t = t_{min} \; MVN\left(\rho_{\varepsilon_w}\varepsilon_w(t-1), \sigma^2_{\varepsilon_w} R(\kappa_w)\right) \; if \; t > t_{min} \right\}$$

S4

where ρ_{ε_n} and ρ_{ε_w} are the estimated degree of autocorrelation in spatio-temporal variation; $R(\kappa_n)$ and $R(\kappa_w)$ are matrices representing the Matérn covariance structure describing the correlation between locations as a function of the decorrelation distance κ_n and κ_w, respectively (Lindgren et al. 2011); and $\sigma^2_{\varepsilon_n}$ and $\sigma^2_{\varepsilon_w}$ are the estimated pointwise variances of spatio-temporal variation.

To estimate all model parameters and calculate derived quantities with associated standard errors, VAST adopts a predictive process approach (Banerjee et al. 2008; Finley et al. 2009) and leverages the TMB R package (Kristensen et al. 2016). The predictive process approach approximates the spatial and spatio-temporal random effect values at every unique sample location using the SPDE estimated over a constant spatial mesh, which is created with the R-INLA R package (Rue et al. 2009; Lindgren et al. 2011). Fixed effects parameters are then estimated by maximizing the marginal likelihood integrated across all random effects, which is approximated using the Laplace approximation as implemented in TMB. Finally, random effects are estimated using an empirical Bayes approach, which identifies the random effects values that maximize the joint likelihood conditional on the fixed effect maximum likelihood parameter estimates. After generating all model parameters, model predicted probability of presence and abundance from equation S1.1 and S1.2 were integrated over a fixed grid with a 25 x 25 km resolution, producing continuous predictions across space for each season-year model timestep.

Spatio-temporal model parameter estimates from best fitted model

For the Environmental Covariates + Spatial + Spatio-Temporal model, which showed the best fit to the data and overall predictive performance (see main text Table) the T-statistic was calculated on the parameter estimates (estimate / standard error) for each linear predictor (LP) to see which parameters were relatively more likely to be statistically significant, and the magnitude of the standard errors (% difference) was calculated to measure which had relatively better precision (less than 30%) (Appendix S1 Table S3). Parameters likely to be the most statistically significant are in bold font, because these statistics are related, these parameters also had relatively better precision (low % difference). Parameters with lower t-statistics but suggestive trends, are in italicized font.

Appendix S1, Table S2: Spatio-temporal model parameter estimates from best fitted model.

LP	Parameter	Est.	Std.Err	t-statistic	Relative Std.Err (%)	Description
1	gamma1_cp	-0.981	0.305	-3.212	-31.136	Summer effect
1	gamma1_cp	-0.821	0.258	-3.180	-31.445	Fall effect
1	gamma1_cp	2.830	0.981	2.883	34.684	Depth effect, First Basis
1	gamma1_cp	-4.327	1.623	-2.667	-37.496	Depth effect, Second Basis
1	*gamma1_cp*	*6.865*	*1.238*	*5.545*	*18.033*	*Bottom Temp effect, First Basis*
1	gamma1_cp	-2.611	0.981	-2.662	-37.569	Bottom Temp effect, Second Basis
1	gamma1_cp	0.849	0.784	1.083	92.300	Surface Temp effect, First Basis
1	gamma1_cp	1.039	0.430	2.417	41.375	Surface Temp effect, Second Basis
1	lambda1_k	0.289	0.266	1.086	92.048	Catchability effect of the DFO survey
1	lambda1_k	1.194	0.612	1.952	51.242	Catchability effect of the NF survey
**1**	**L_omega1_z**	**3.446**	**0.457**	**7.543**	**13.258**	**Spatial variation, persistent spatial patterns not explained by covariates**
**1**	**L_epsilon1_z**	**0.137**	**0.018**	**7.795**	**12.828**	**Spatio-temporal variability, spatial patterns shift across years**
1	L_beta1_z	-0.076	0.026	-2.887	-34.635	Intercept, persistence and drift across years
**1**	**logkappa1**	**-4.594**	**0.132**	**-34.926**	**-2.863**	**log of the Spatial range, scale of the spatial autocorrelation, decorrelation rate**
**1**	**Beta_mean1_c**	**-7.131**	**1.129**	**-6.318**	**-15.829**	**Mean temporal variation**
2	*gamma2_cp*	*0.838*	*0.196*	*4.265*	*23.448*	*Summer effect*
2	*gamma2_cp*	*0.551*	*0.165*	*3.335*	*29.989*	*Fall effect*
2	gamma2_cp	-1.534	0.582	-2.637	-37.923	Depth effect, First Basis
2	gamma2_cp	1.986	1.066	1.864	53.658	Depth effect, Second Basis
2	gamma2_cp	0.423	0.912	0.464	215.358	Bottom Temp effect, First Basis
2	gamma2_cp	-0.308	0.624	-0.493	-202.691	Bottom Temp effect, Second Basis
2	gamma2_cp	-1.213	0.499	-2.430	-41.156	Surface Temp effect, First Basis
2	*gamma2_cp*	*-1.423*	*0.275*	*-5.168*	*-19.350*	*Surface Temp, Second Basis*
2	lambda2_k	0.192	0.202	0.947	105.579	Catchability effect of the DFO Maritimes survey
2	lambda2_k	-0.270	0.243	-1.114	-89.734	Catchability effect of the DFO Newfoundland survey
**2**	**L_omega2_z**	**0.892**	**0.072**	**12.403**	**8.063**	**Spatial variation, persistent spatial patterns not explained by covariates**
**2**	**L_epsilon2_z**	**-0.699**	**0.035**	**-19.716**	**-5.072**	**Spatio-temporal variability, spatial patterns shift across years**
2	*L_beta2_z*	*0.067*	*0.020*	*3.366*	*29.707*	*Intercept, persistence and drift across years*
**2**	**logkappa2**	**-2.975**	**0.090**	**-33.061**	**-3.025**	**log of the spatial range, scale of the spatial autocorrelation, decorrelation rate**
2	*Beta_mean2_c*	*2.603*	*0.451*	*5.770*	*17.330*	*Mean temporal variation*
**2**	**Beta_rho2_f**	**0.985**	**0.029**	**34.100**	**2.933**	**Degree of autocorrelation in temporal variation**
2	Epsilon_rho2_f	0.209	0.171	1.222	81.851	Degree of autocorrelation in spatio-temporal variation

Parameter Estimates, T- Statistics, and magnitude of the standard errors calculated from the parameter estimate standard deviation report from the Environmental Covariates + Spatial + Spatio-Temporal model. Parameters likely to be the most statistically significant are in bold font, and parameters with lower t-statistics but suggestive trends, are shaded in italicized font.

Abbreviations: Linear Predictor (LP), Estimate (Est.), Standard Error (Std.Err), Department of Fisheries and Oceans Canada (DFO).

Appendix S1, Table S3: Explanation of focal area delegation and supporting evidence.

Focal Area	Evidence	Details
Cape Cod	1h, 5	Including the offshore waters of Cape Cod, Massachusetts Bay, and north to include Jeffreys Ledge. A region where Atlantic halibut were once so abundant that they were considered a nuisance to Cod fishermen(Grasso, 2008). Cape Cod, inshore, has been clipped outside of the study area due to shallow depths.
Nantucket Shoals	1h, 5	Waters surrounding Nantucket Island, and a historical fishing ground, a portion of this focal area has been clipped outside of the study area due to shallow depths.
Georges Bank	1h, 4, 5	Covering the Geological area of the Georges Bank, this focal area extends across the Hague line and represents a transboundary component of the population, and a historically fruitful fishing ground.
Eastern Gulf of Maine	2, 5	Extending along the coast of Maine, across Jordan Basin, to the Hague line/ Bay of Fundy as it pertains to transboundary discussion .
Bay of Fundy	2, 4, 5	Maritimes Region RV survey strata 490-495 extending the southernmost limit straight across to the Hague line and the Eastern Gulf of Maine as it pertains to transboundary discussion (DFO 2023). The southern part of this is associated with persistently high abundance.
Browns Bank	2, 3, 4, 5	From DFO's Ecological and biologically significant areas and extended to the south shore of Nova Scotia to capture a region with persistently high abundance.
Sable Island (west)	3, 4	Includes Middle Bank, Western Bank, and the western portion of Sable Island bank. A region with persistently high abundance.
Gully	1c, 3, 4	Extends around the Gully MPA, and across the western portion of Banquereau Bank to capture a region with persistently high abundance.
Cape Breton	1c	Represents NAFO area 4Vn, this is a recently developing area of significance to the fishery. This is also an area that is well known for seasonal changes in groundfish abundance and connectivity to the Gulf of St. Lawrence.
Halibut Channel	3,1h	Historically important for halibut commercial landings and location of high juvenile abundance in 1978-1989, but not since.

Southern Grand Banks	1c,1h	The shelf edge is important fishing grounds which captures larger halibut. Seasonal shift to deeper water in this area for spawning and foraging.
Grand Banks Tail	1c, 1h	NAFO regulated waters with commercial halibut catches on the shelf edge. Seasonal shift to deeper water in this area for spawning and foraging.

Evidence Key:
1h. Historical records of fisheries (McCracken 1958; Grasso 2008).
1c. Current records of fisheries (den Heyer et al. 2012; Ransier et al. 2024) and of fleet approach descriptions (Cox 2016).
2. PSAT and conventional tagging data (Ransier et al. 2024).
3. Spatio-temporally model showing persistent areas of high juvenile density over 3-4 decades(Boudreau et al. 2017).
4. A strong relationship between the size of juvenile habitat and adult landings (French et al. 2018).
5. A gradient in life history traits: fish maturing at smaller sizes in the southern range (Shackell et al. 2019).

To represent the spatial stock structure of Atlantic halibut (*Hippoglossus hippoglossus*), focal areas were identified and drawn within the study area. Delegations were based on five sources of evidence relating to stock structure and historical and current distributions in the targeted fishery (listed under “key evidence”).

Abbreviations: Research Vessel (RV), The Department of Fisheries and Oceans Canada (DFO), Marine Protected Area (MPA), North Atlantic Fisheries Organization (NAFO).

Appendix S1, Table S4: Centre of Gravity locations for each region and focal area.

Region/Focal Area	Period	Long.Start	Lat.Start	Long. End	Lat.End	Distance (km)	Distance Per Year
Canada	1990-2005	-61.59	43.80	-62.09	43.84	40.72	2.71
Canada	2006-2023	-61.95	43.90	-59.55	44.35	198.40	11.67
USA	1990-2005	-68.64	42.77	-68.46	43.46	77.71	5.18
USA	2006-2023	-68.35	43.52	-68.62	43.23	39.86	2.34
Nantucket	1990-2005	-69.31	40.96	-69.25	41.14	20.82	1.39
Nantucket	2006-2023	-69.28	41.14	-69.29	41.12	2.22	0.13
Georges	1990-2005	-66.48	41.99	-66.48	41.99	0.89	0.06
Georges	2006-2023	-66.52	41.86	-66.55	41.94	8.58	0.50
CapeCod	1990-2005	-69.84	42.77	-69.87	42.89	13.86	0.92
CapeCod	2006-2023	-69.91	42.82	-69.96	42.77	6.97	0.41
EGOM	1990-2005	-68.33	43.82	-68.26	43.87	7.70	0.51
EGOM	2006-2023	-68.16	43.94	-68.26	43.88	10.87	0.64
BOF	1990-2005	-66.47	44.36	-66.55	44.30	8.92	0.59
BOF	2006-2023	-66.51	44.34	-66.41	44.43	13.42	0.79
Browns	1990-2005	-65.60	42.68	-65.71	42.74	10.95	0.73
Browns	2006-2023	-65.68	42.74	-65.63	42.77	4.97	0.29
Sable	1990-2005	-61.97	43.46	-61.91	43.44	5.04	0.34

Region/Focal Area	Period	Long.Start	Lat.Start	Long. End	Lat.End	Distance (km)	Distance Per Year
Sable	2006-2023	-61.79	43.46	-61.75	43.61	16.66	0.98
Gully	1990-2005	-59.39	43.87	-59.37	44.03	18.39	1.23
Gully	2006-2023	-59.51	43.93	-59.30	44.12	26.66	1.57
CapeBreton	1990-2005	-59.33	46.60	-59.27	46.45	16.60	1.11
CapeBreton	2006-2023	-59.38	46.55	-59.29	46.39	19.24	1.13
GrandBanks	1990-2005	-53.04	44.25	-53.14	44.35	14.08	0.94
GrandBanks	2006-2023	-53.27	44.46	-53.16	44.41	9.82	0.58
GBTail	1990-2005	-49.92	43.44	-49.89	43.39	5.95	0.40
GBTail	2006-2023	-49.88	43.40	-49.98	43.39	8.74	0.51
HaliChan	1990-2005	-56.15	45.42	-56.11	45.35	8.86	0.59
HaliChan	2006-2023	-56.24	45.31	-56.00	45.46	24.67	1.45

Starting and ending longitude and latitudes of Centre of Gravity for all regions and focal areas in two periods 1990-2005 and 2006-2023. Distance (km) represents the distance between the corresponding latitudinal longitudinal coordinates using the great circle equation.

Abbreviations: Longitude (Lon.), Latitude (Lat.), United States of America (USA), Nantucket Shoals (Nantucket), Georges Bank (Georges), Cape Cod (CapeCod), Eastern Gulf of Maine (EGOM), Bay of Fundy (BOF), Browns Bank (Browns), Sable Island Bank (Sable), Cape Breton (CapeBreton), Grand Banks (GrandBanks), Tail of the Grand Banks (GBTail), Halibut Channel (HaliChan)

References

Banerjee, Sudipto, Alan E. Gelfand, Andrew O. Finley, and Huiyan Sang. 2008. “Gaussian Predictive Process Models for Large Spatial Data Sets.” *Journal of the Royal Statistical Society Series B: Statistical Methodology* 70 (4): 825–48. https://doi.org/10.1111/j.1467-9868.2008.00663.x.

Boudreau, Stephanie A., Nancy L. Shackell, Stuart Carson, and Cornelia E. den Heyer. 2017. “Connectivity, Persistence, and Loss of High Abundance Areas of a Recovering Marine Fish Population in the Northwest Atlantic Ocean.” *Ecology and Evolution* 7 (22): 9739–49. https://doi.org/10.1002/ece3.3495.

Charsley, Anthony R., Arnaud Grüss, James T. Thorson, et al. 2023. “Catchment-Scale Stream Network Spatio-Temporal Models, Applied to the Freshwater Stages of a Diadromous Fish Species, Longfin Eel (*Anguilla Dieffenbachii*).” *Fisheries Research* 259 (March): 106583. https://doi.org/10.1016/j.fishres.2022.106583.

Claytor, R, D Clark, T McIntyre, et al. 2014. *Review of Surveys Contributing to Groundfish Assessments with Recommendations for an Ecosystem Survey Program in the Maritimes Region*. Canadian Technical Report of Fisheries and Aquatic Sciences, no. 3083: : x + 82 p.

Cox, S. P. 2016. “Framework for the Assessment of Atlantic Halibut Stocks on the Scotian Shelf and Southern Grand Banks.” . . *DFO Can. Sci. Advis. Sec. Res. Doc.* 2016/001. v + 57p.

DFO. 2023. *Maritimes Research Vessel Survey Trends on the Scotian Shelf and Bay of Fundy for 2022*. Science Response 2023/045.

DFO. 2024. *Newfoundland & Labrador Comparative Fishing Analysis – Part 1*. No. 2024/002. Canadian Science Advisory Secretariat Science Advisory Report. DFO Fisheries and Oceans Canada. https://publications.gc.ca/collections/collection_2024/mpo-dfo/fs70-6/Fs70-6-2024-002-eng.pdf.

Finley, Andrew O., Huiyan Sang, Sudipto Banerjee, and Alan E. Gelfand. 2009. “Improving the Performance of Predictive Process Modeling for Large Datasets.” *Computational Statistics & Data Analysis* 53 (8): 2873–84. https://doi.org/10.1016/j.csda.2008.09.008.

French, Kiyomi J., Nancy L. Shackell, and Cornelia E. den Heyer. 2018. “Strong Relationship between Commercial Catch of Adult Atlantic Halibut (*Hippoglossus Hippoglossus*) and Availability of Suitable Habitat for Juveniles in the Northwest Atlantic Ocean.” *Fishery Bulletin* 116 (2): 111–31.

Grasso, Glenn M. 2008. “What Appeared Limitless Plenty: The Rise and Fall of the Nineteenth-Century Atlantic Halibut Fishery.” *Environmental History* 13 (1): 66–91.

Heyer, C. E. den, A. Armsworthy, S. Wilson, et al. 2012. “Atlantic Halibut All-Sizes Tagging Program Summary Report for 2006 to 2011.” *Canadian Technical Report of Fisheries and Aquatic Sciences* 2992: 18–23.

Kristensen, Kasper, Anders Nielsen, Casper W. Berg, Hans Skaug, and Bradley M. Bell. 2016. "TMB: Automatic Differentiation and Laplace Approximation." *Journal of Statistical Software* 70: 1–21. https://doi.org/10.18637/jss.v070.i05.

Lindgren, Finn, Håvard Rue, and Johan Lindström. 2011. "An Explicit Link between Gaussian Fields and Gaussian Markov Random Fields: The Stochastic Partial Differential Equation Approach." *Journal of the Royal Statistical Society Series B: Statistical Methodology* 73 (4): 423–98. https://doi.org/10.1111/j.1467-9868.2011.00777.x.

Maureaud, A. A., R. Frelat, L. Pecuchet, et al. 2021. "Are We Ready to Track Climate-Driven Shifts in Marine Species across International Boundaries? - A Global Survey of Scientific Bottom Trawl Data." *Global Change Biology* 27 (2): 220–36; https://doi.org/10.1111/GCB.15404. https://doi.org/10.1111/gcb.15404.

McCracken, F. D. 1958. "On the Biology and Fishery of the Canadian Atlantic Halibut, *Hippoglossus Hippoglossus*, L." *Journal of the Fisheries Board of Canada* 15 (6): 1269–311.

Miller, Timothy J., Chhandita Das, Philip Politis, et al. 2010. *Estimation of Albatross IV to Henry B. Bigelow Calibration Factors*. Northeast Fisheries Science Center Reference Document ; 10-05. Northeast Fisheries Science Center (U.S.). :/view/noaa/3726.

Ransier, Krista T., Paul Gatti, Arnault Le Bris, et al. 2024. "Electronic Tags Reveal High Migratory Diversity within the Largest Atlantic Halibut (Hippoglossus Hippoglossus) Stock." *Canadian Journal of Fisheries and Aquatic Sciences* 81 (7): 828–46. https://doi.org/10.1139/cjfas-2023-0282.

Rue, Havard, and Leonhard Held. 2005. *Gaussian Markov Random Fields: Theory and Applications*. Chapman and Hall/CRC. https://doi.org/10.1201/9780203492024.

Rue, Håvard, Sara Martino, and Nicolas Chopin. 2009. "Approximate Bayesian Inference for Latent Gaussian Models by Using Integrated Nested Laplace Approximations." *Journal of the Royal Statistical Society Series B: Statistical Methodology* 71 (2): 319–92. https://doi.org/10.1111/j.1467-9868.2008.00700.x.

Shackell, Nancy L., Kiyomi J. Ferguson, Cornelia E. den Heyer, David Brickman, Zeliang Wang, and Krista T. Ransier. 2019. "Growing Degree-Day Influences Growth Rate and Length of Maturity of Northwest Atlantic Halibut (*Hippoglossus Hippoglussus* L.) across the Southern Stock Domain." *Journal of Northwest Atlantic Fishery Science* 50: 25–35.

Thorson, James T. 2019. "Guidance for Decisions Using the Vector Autoregressive Spatio-Temporal (VAST) Package in Stock, Ecosystem, Habitat and Climate Assessments." *Fisheries Research* 210: 143–61.

Thorson, James T., Mark D. Scheuerell, Andrew O. Shelton, Kevin E. See, Hans J. Skaug, and Kasper Kristensen. 2015. "Spatial Factor Analysis: A New Tool for Estimating Joint Species Distributions and Correlations in Species Range." *Methods in Ecology and Evolution* 6 (6): 627–37. https://doi.org/10.1111/2041-210X.12359.
