# Supplementary material for: Shifts and expansion in a spatially structured fish stock spanning international borders in the Northwest Atlantic
Source: Ecol Appl. 2026 Sep 16;36(6):e70311. doi: 10.1002/eap.70311 (PMC13581450; doi:10.1002/eap.70311)
Supplement: Supplementary file 2 [file EAP-36-e70311-s002.pdf]

Appendix S2

Shifts and expansion in a spatially structured fish stock spanning international borders in the Northwest Atlantic

Nancy L Shackell, Kiyomi J Ferguson, Andrew J. Allyn, Cornelia E. den Heyer, Jamie C. Tam, David M Keith, Katherine E. Mills, James T. Thorson

Ecological Applications

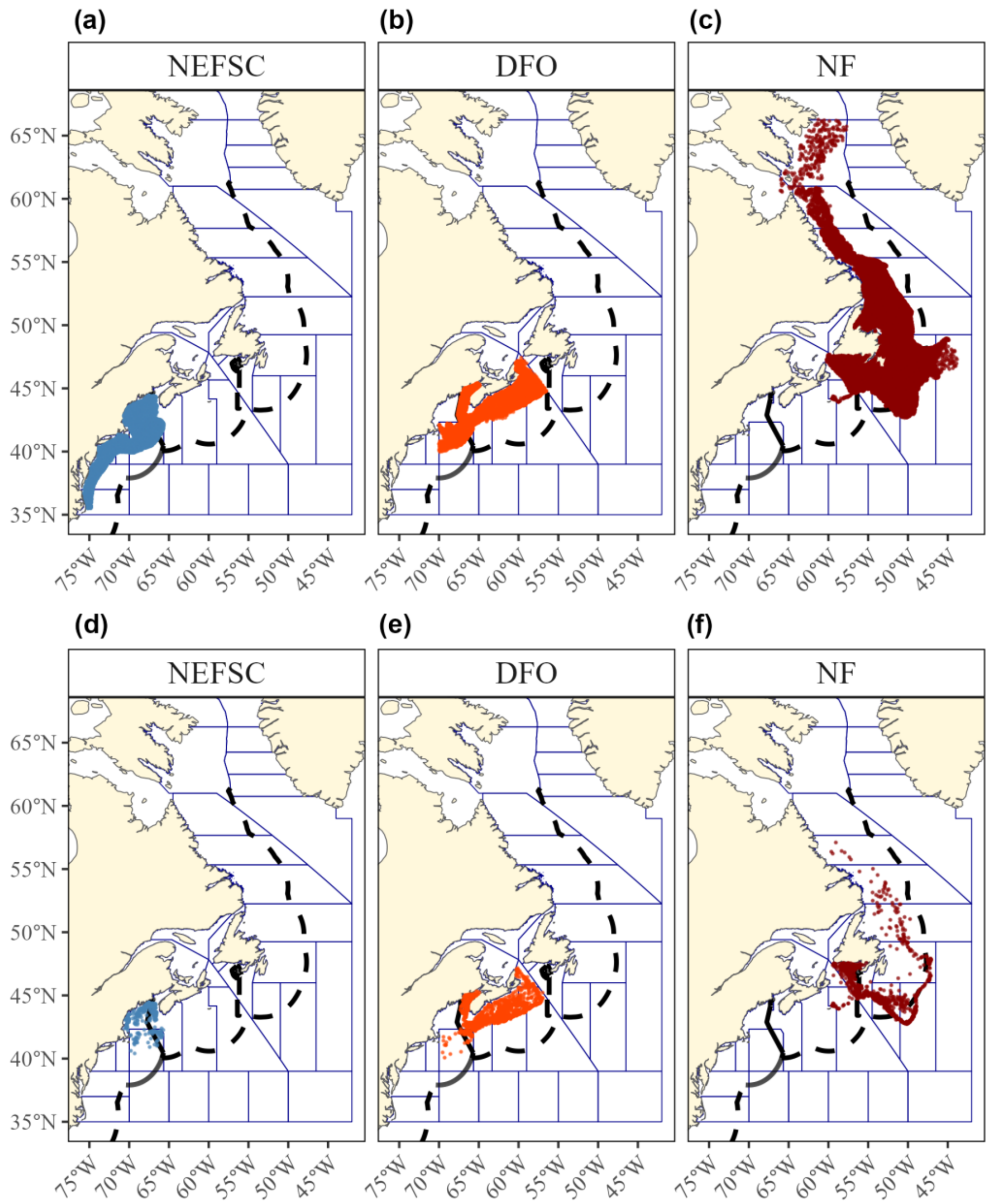

Appendix S2: Figure S1. Survey locations by regulatory agency and halibut presence. Regulatory agencies include: the National Oceanic and Atmospheric Administration's (NOAA) Northeast Fisheries Science Center (NEFSC), Department of Fisheries and Oceans Canada (DFO) Maritimes, and DFO Newfoundland (NF) research vessel surveys. Top row: All survey trawl locations, 1990-2023: (a)NEFSC, (b)DFO Maritimes, (c)DFO Newfoundland. Bottom row: all survey trawl locations with Atlantic halibut (*Hippoglossus hippoglossus*) presence, 1990-2023, (d) NEFSC, (e) DFO Maritimes, (f) DFO Newfoundland.

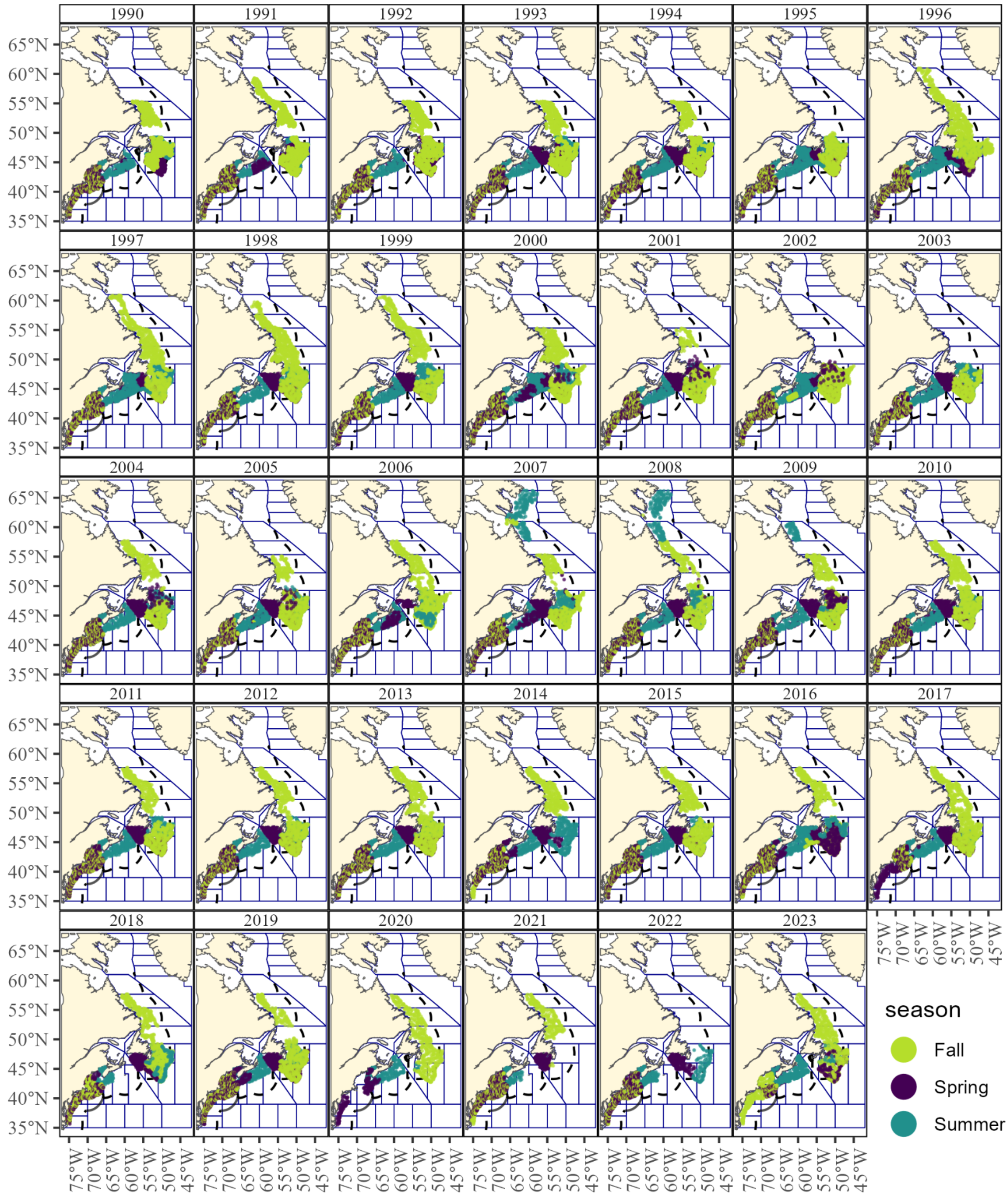

Appendix S2: Figure S2. Annual distribution of survey locations (1990-2023). Including data from the National Oceanic and Atmospheric Administration's (NOAA) Northeast Fisheries Science Center (NEFSC), Department of Fisheries and Oceans Canada (DFO) Maritimes, and DFO Newfoundland (NF), spring, summer, and fall research vessel surveys.

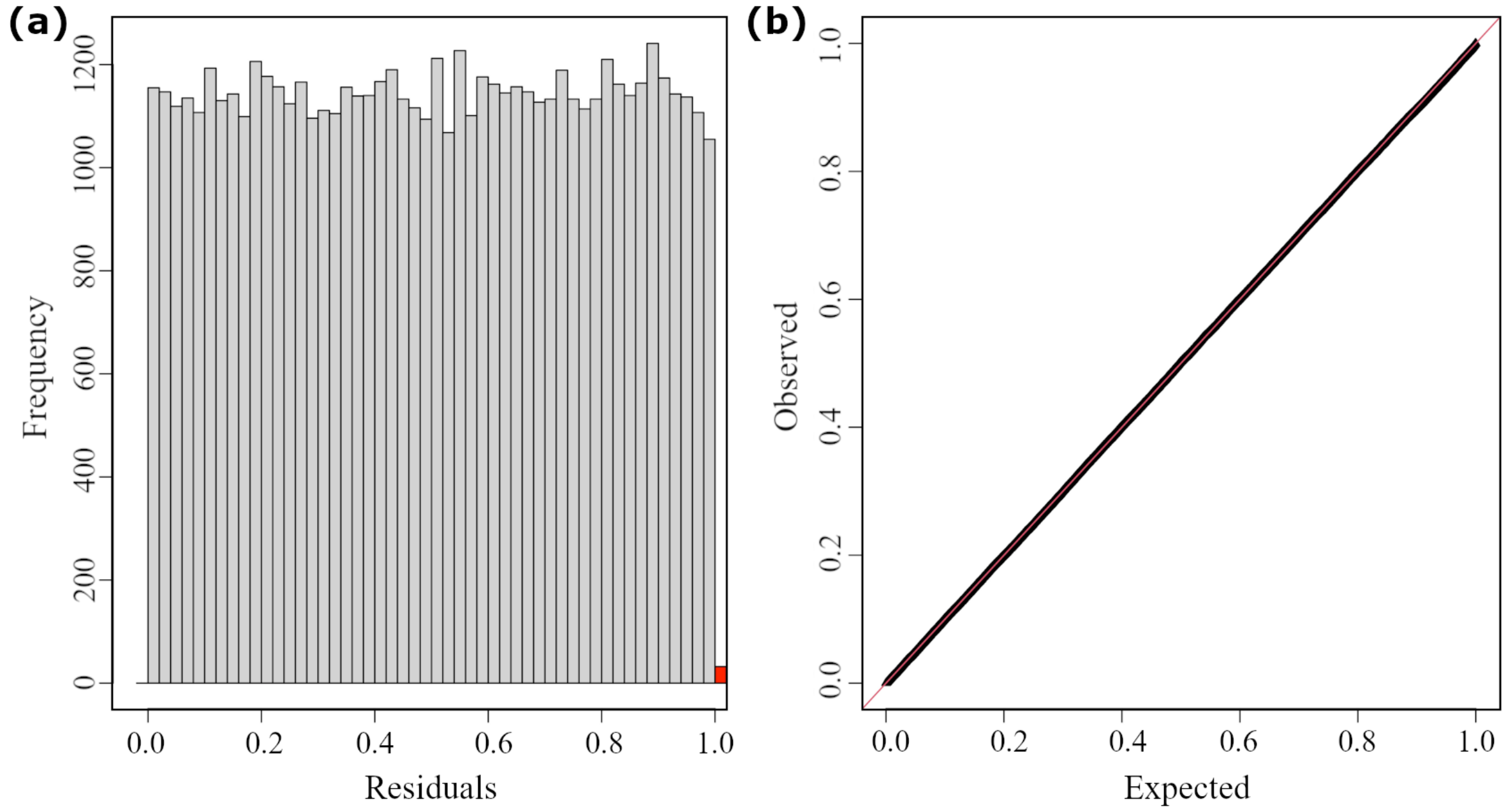

Appendix S2: Figure S3. Residual diagnostic plots produced using the R package "DHARMa" (Harting 2024): (a) histogram of residuals, (b) QQ plot of residuals, for the Environment + Spatial + Spatio-temporal Model.

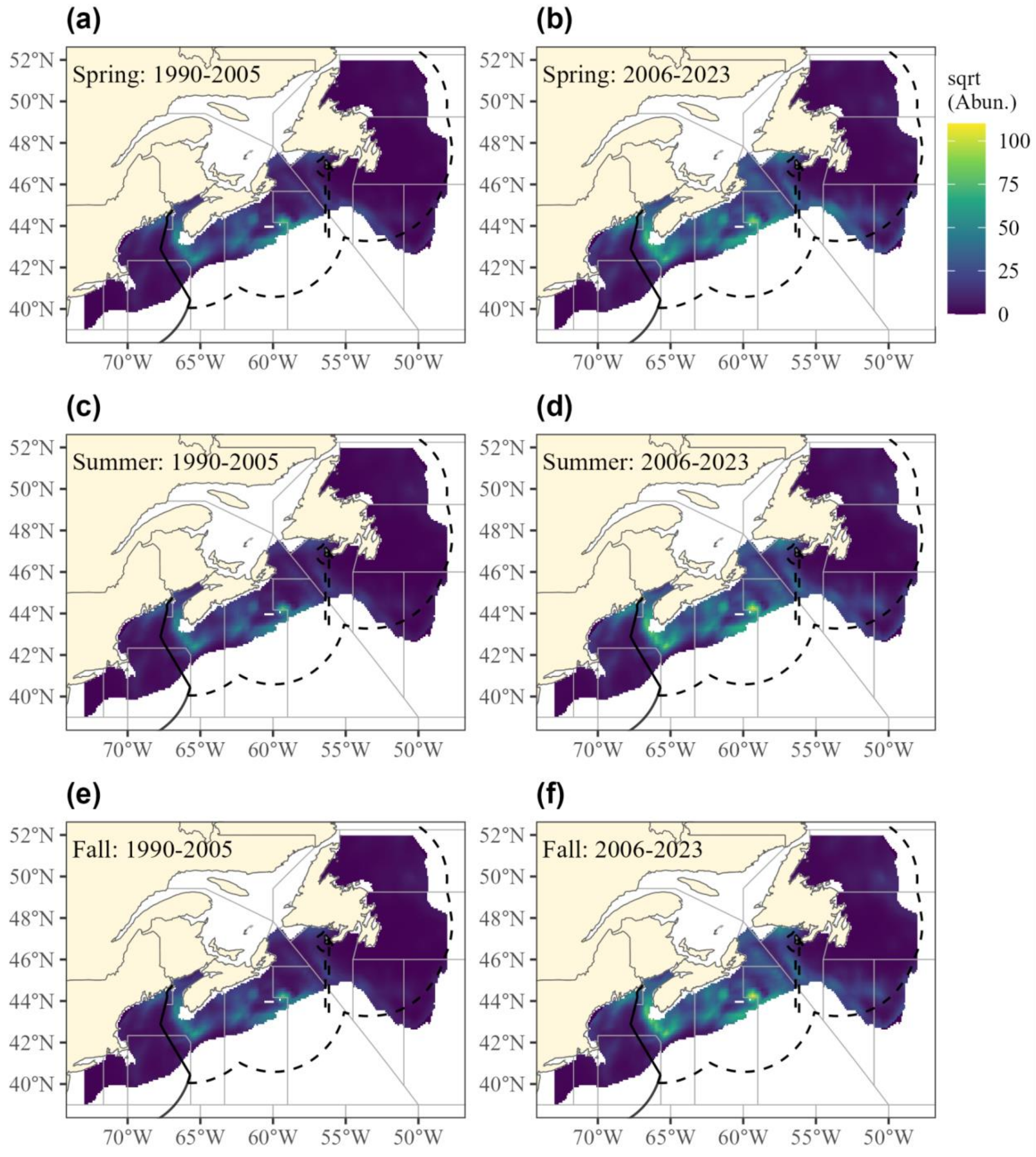

Appendix S2: Figure S4. Comparison of modeled abundance for Atlantic halibut (*Hippoglossus hippoglossus*) both seasonally, and between two timeframes: 1990-2005 and 2006- 2023, the latter representing a period of accelerated warming. (a) Spring 1990-2005, (b) Spring 2006- 2023, (c) Summer 1990-2005, (d) Summer 2006- 2023, (e) Fall 1990-2005, (f) Fall 2006- 2023. Across seasons, spatial patterns of estimated abundance appear consistent between timeframes. Abundance estimates were square-root transformed and interpolated using bivariate linear interpolation (Akima and Gebhardt 1998) for mapping purposes. Winter estimates are unavailable; the season was excluded from the analysis because most regions do not conduct surveys at that time of year.

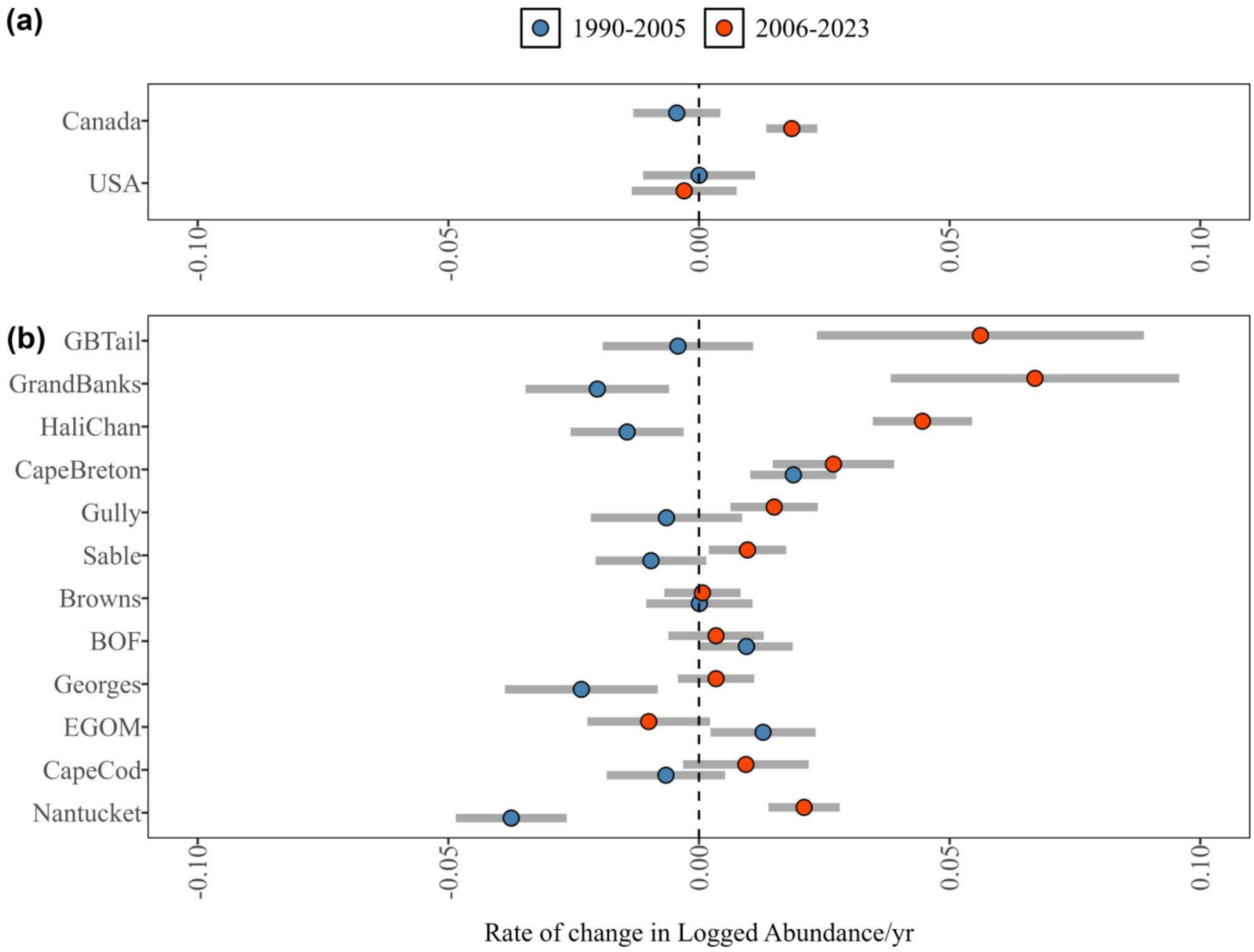

Appendix S2: Figure S5. Slope and confidence interval (CI) of logged abundance over time within two periods, 1990-2005 (blue) and 2006-2023 (red). Slopes are centred between positive (increasing) and negative(decreasing) trends, if the CI for a slope includes zero (indicated in plots with black dashed line), it is not statistically significant. (a) Regions: Canada and USA (b) Focal areas, arranged roughly north to south: Tail of the Grand Banks (GBTail), Grand Banks (GrandBanks), Halibut Channel (HaliChan), Cape Breton (CapeBreton), Gully (Gully), Sable Island Bank (Sable), Browns Bank (Browns), Bay of Fundy (BOF), Georges Bank (Georges), Eastern Gulf of Maine (EGOM), Cape Cod (CapeCod), Nantucket (Nantucket Shoals).

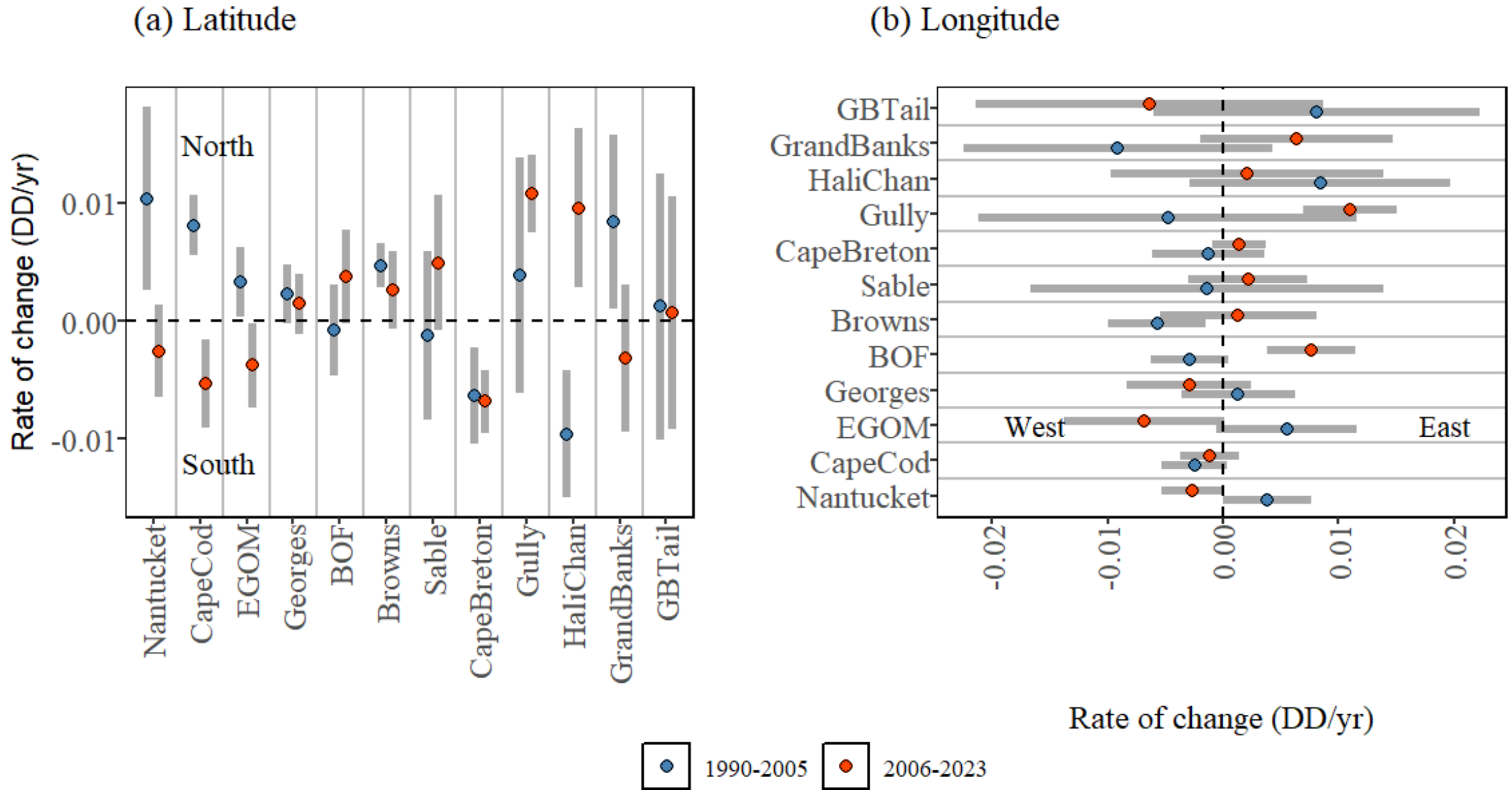

Appendix S2: Figure S6. Slope and confidence interval (CI) of the Centre of Gravity (COG) over time within two periods, 1990-2005 (blue) and 2006-2023 (red), and in 12 focal areas. Focal areas are arranged roughly west to east: Nantucket refers to Nantucket Shoals, Georges Bank (Georges), Cape Cod (CapeCod), Eastern Gulf of Maine (EGOM), Bay of Fundy (BOF), Browns Bank (Browns), Sable Island Bank (Sable), Gully (Gully), Cape Breton (CapeBreton), Halibut Channel (HaliChan), Grand Banks (GrandBanks), Tail of the Grand Banks (GBTail). If the CI for a slope includes zero (indicated in plots with black dashed line), it is not statistically significant. (a) Latitude: Slopes are centred between positive (northward) and negative (southward) COG movement. (b) Longitude: Slopes are centred between positive (eastward) and negative (westward) COG movement.

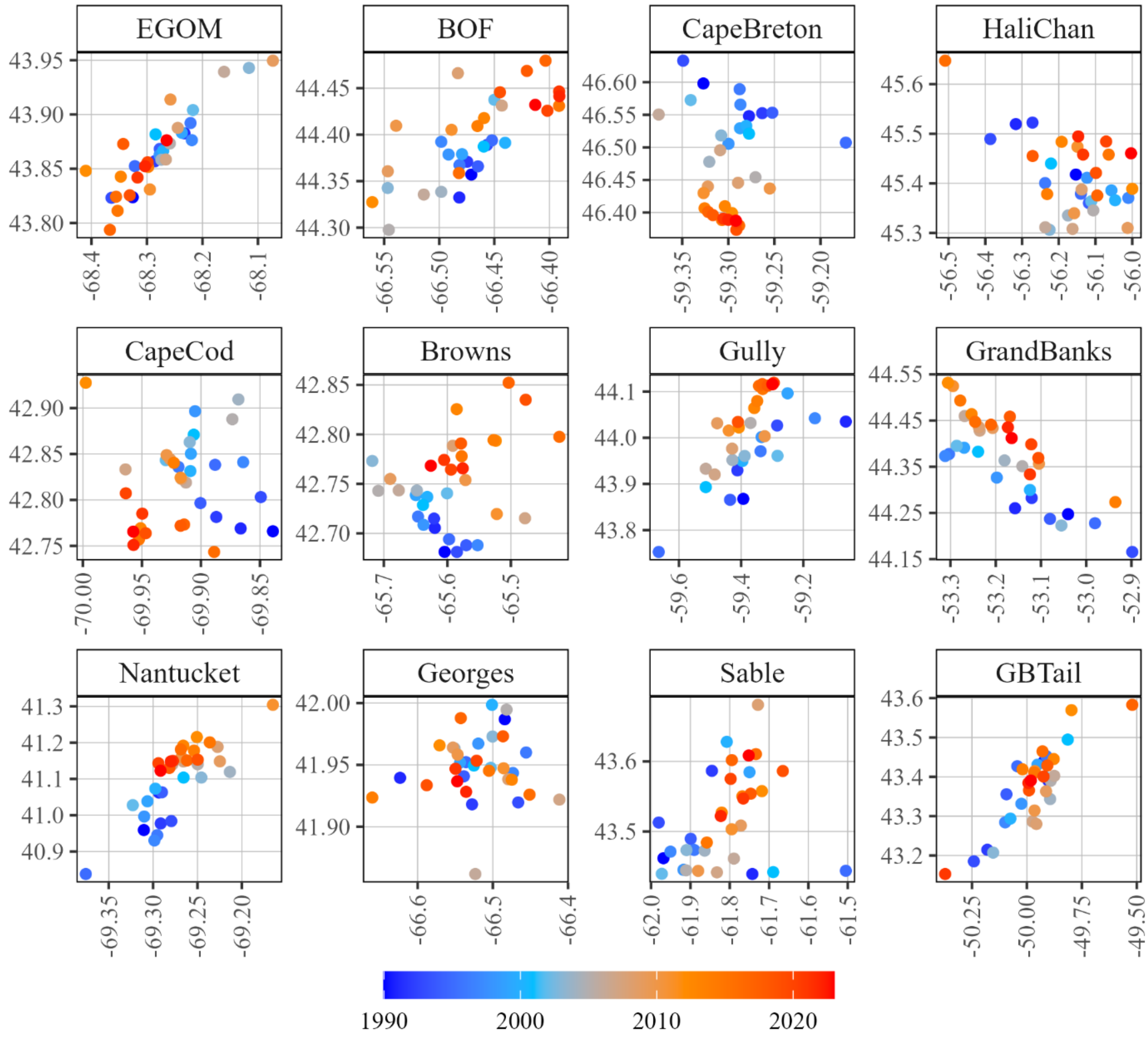

Appendix S2: Figure S7. Annual Centre of Gravity (COG: the average latitude and longitude, weighted by modeled abundance estimates) for each focal area (1990-2023). Plot areas represent the latitudinal (y-axis) and longitudinal (x-axis) range of each focal area's COG. Color gradient represents year, from the beginning of the time series in blue, to the end of the timeseries in red. Focal areas are arranged roughly from west to east: Eastern Gulf of Maine (EGOM), Bay of Fundy (BOF), Cape Breton (CapeBreton), Halibut Channel (HaliChan), Cape Cod (CapeCod), Browns Bank (Browns), Gully (Gully), Grand Banks (GrandBanks), Nantucket Shoals (Nantucket), Georges Bank (Georges), Sable Island Bank (Sable), Tail of the Grand Banks (GBTail).

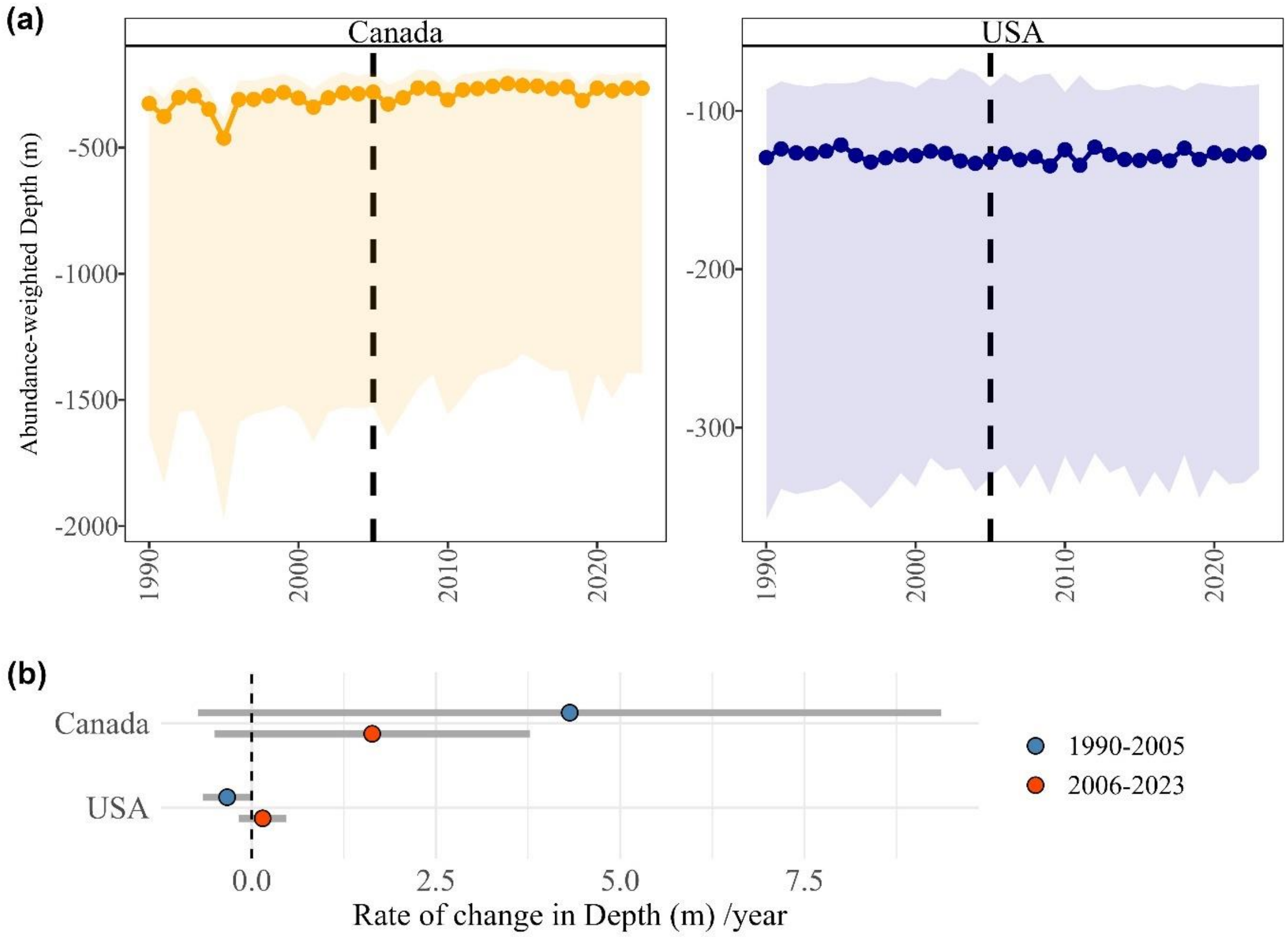

Appendix S2: Figure S8. Regional Abundance-weighted Depth(AWD): (a) Trends in AWD in Canada and the USA from 1990-2023, dashed vertical line at 2005 representing the partition between the two time periods in: (b) Rate of change (m/year) indicated by the slope and confidence interval (CI) of the AWD during 1990-2005 (blue) and 2006-2023 (red). Slopes are centred between positive (shallowing) and negative (deepening) trends, if the CI for a slope includes zero (indicated in plots with black dashed line), it is not statistically significant.

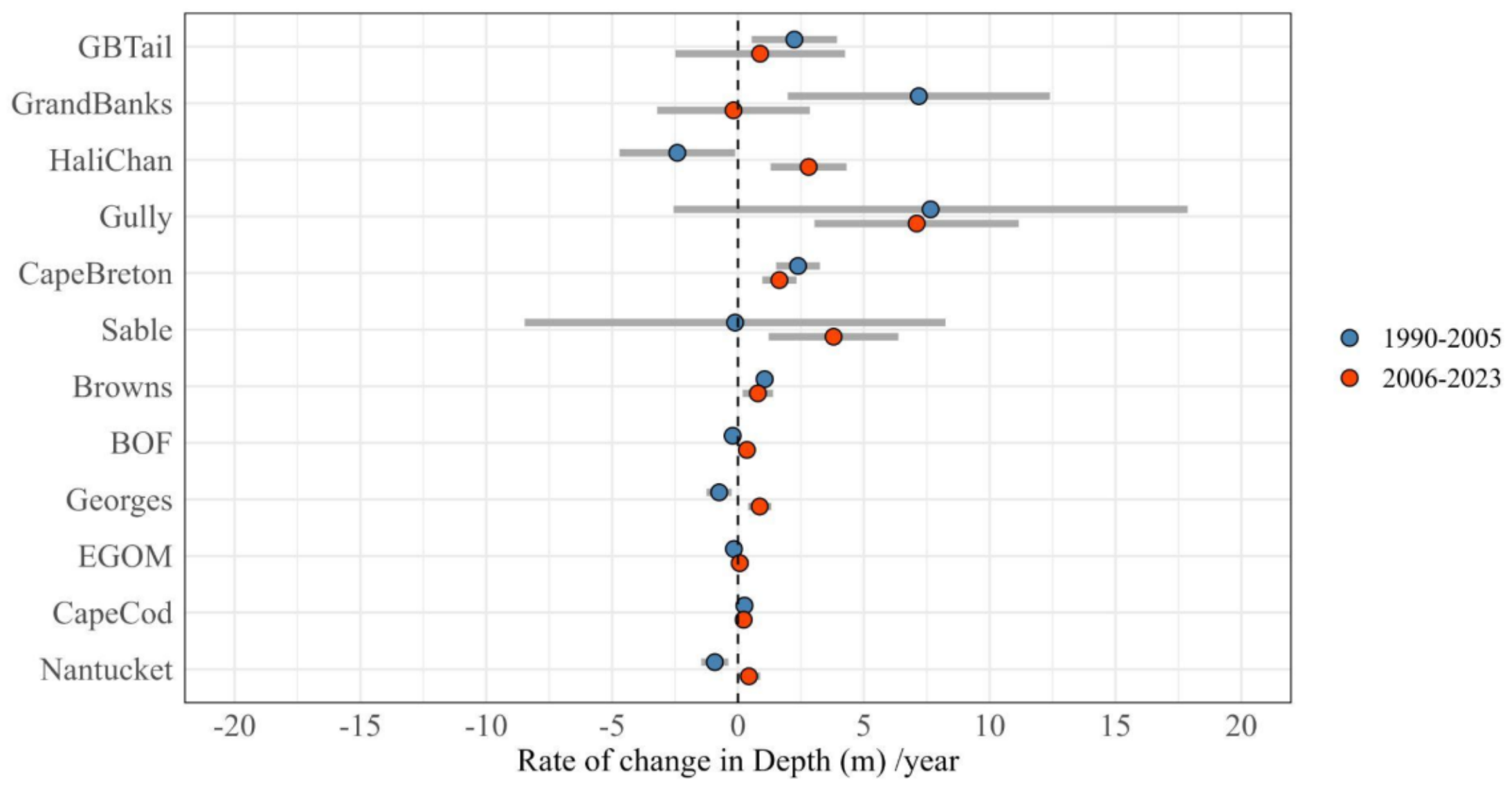

Appendix S2: Figure S9. Rate of change in Abundance-weighted Depth (AWD), m/year indicated by the slope and confidence interval (CI) of the AWD during 1990-2005 (blue) and 2006-2023 (red), for each focal area: Nantucket refers to Nantucket Shoals, Georges Bank (Georges), Cape Cod (CapeCod), Eastern Gulf of Maine (EGOM), Bay of Fundy (BOF), Browns Bank (Browns), Sable Island Bank (Sable), Gully (Gully), Cape Breton (CapeBreton), Halibut Channel (HaliChan), Grand Banks (GrandBanks), Tail of the Grand Banks (GBTail). Gully, Cape Breton, and Grand Banks Tail shallowed at similar rates during both time periods of around ~8 , ~2-2.5, and ~1-3 m/year, respectively. Grand Banks' AWD shallowed at a rate of ~8m/year during 1990-2005 and stabilized during 2006-2023. AWD for Halibut Channel shallowed at a rate ~3m/year from 2006-2023, in contrast to a deepening trend of ~3m/year before (Appendix S2 Figure S9). When it comes to Atlantic halibut, juveniles are typically found in shallower waters (Cargnelli et al. 1999), so a shallowing trend could be reflective of either increased recruitment, or immigration/ emigration leading to a decrease in median age. Variable trends in Newfoundland focal areas (Grand Banks Tail: slight and steady shallowing (~1-3m/year) in both timeframes, Grand Banks: shallowing ~8m/year (1990-2005), and Halibut Channel: shallowing ~2.5m/year (2006-2023), could reflect the return of a core area of persistently high abundance which faded away in the early 1990's (Boudreau et al. 2017). The steady shallowing trend in the Gully and Cape Breton, where the increase in juvenile abundance has been largest, supports the hypothesis that the shift to more shallow waters reflects changes in the age structure of the population.

References

Akima, Hiroshi, and Albrecht Gebhardt. 1998. "Akima: Interpolation of Irregularly and Regularly Spaced Data." CRAN: Contributed Packages. The R Foundation, August 20. https://doi.org/10.32614/cran.package.akima.

Boudreau, Stephanie A., Nancy L. Shackell, Stuart Carson, and Cornelia E. den Heyer. 2017. "Connectivity, Persistence, and Loss of High Abundance Areas of a Recovering Marine Fish Population in the Northwest Atlantic Ocean." *Ecology and Evolution* 7 (22): 9739–49. https://doi.org/10.1002/ece3.3495.

Cargnelli, Luca M., Sara J. Griesbach, and Wallace W. Morse. 1999. *Atlantic Halibut,* Hippoglossus Hippoglossus*, Life History and Habitat Characteristics. Essential Fish Habitat Source Document*. NMFS-NE-125: 1–17.

Harting, Florian. 2024. *DHARMa: Residual Diagnostics for Hierarchical (Multi-Level/Mixed) Regression Models*. V. R package version 0.4.7. Theoretical Ecology, released October 17. https://cran.r-project.org/web/packages/DHARMa/vignettes/DHARMa.html.
